# Importance of endothelial *Hey1* expression for thoracic great vessel development and its distal enhancer for Notch-dependent endothelial transcription

**DOI:** 10.1074/jbc.RA120.015003

**Published:** 2020-10-16

**Authors:** Yusuke Watanabe, Daiki Seya, Dai Ihara, Shuhei Ishii, Taiki Uemoto, Atsushi Kubo, Yuji Arai, Yoshie Isomoto, Atsushi Nakano, Takaya Abe, Mayo Shigeta, Teruhisa Kawamura, Yoshihiko Saito, Toshihiko Ogura, Osamu Nakagawa

**Affiliations:** 1 Department of Molecular Physiology, National Cerebral and Cardiovascular Center Research Institute, Suita, Osaka, Japan; 2 Graduate School of Medical Sciences, Nara Medical University, Kashihara, Nara, Japan; 3 Laboratory of Stem Cell and Regenerative Medicine, Department of Biomedical Sciences, College of Life Sciences, Ritsumeikan University, Kusatsu, Shiga, Japan; 4 Department of Developmental Neurobiology, Institute of Development, Aging, and Cancer, Tohoku University, Sendai, Miyagi, Japan; 5 Laboratory of Animal Experiment and Medical Management, National Cerebral and Cardiovascular Center Research Institute, Suita, Osaka, Japan; 6 Laboratory for Animal Resources and Genetic Engineering, RIKEN Center for Biosystems Dynamics Research, Kobe, Japan; 7 Department of Cardiovascular Medicine, Nara Medical University, Kashihara, Nara, Japan

**Keywords:** Hey1, pharyngeal arch artery, great vessel morphogenesis, development, cardiovascular disease, transcription regulation, gene knockout, embryo, endothelial cell, great vessel morphogenesis, Hey1, Notch signaling

## Abstract

Thoracic great vessels such as the aorta and subclavian arteries are formed through dynamic remodeling of embryonic pharyngeal arch arteries (PAAs). Previous work has shown that loss of a basic helix-loop-helix transcription factor Hey1 in mice causes abnormal fourth PAA development and lethal great vessel anomalies resembling congenital malformations in humans. However, how Hey1 mediates vascular formation remains unclear. In this study, we revealed that Hey1 in vascular endothelial cells, but not in smooth muscle cells, played essential roles for PAA development and great vessel morphogenesis in mouse embryos. *Tek-Cre*–mediated *Hey1* deletion in endothelial cells affected endothelial tube formation and smooth muscle differentiation in embryonic fourth PAAs and resulted in interruption of the aortic arch and other great vessel malformations. Cell specificity and signal responsiveness of *Hey1* expression were controlled through multiple *cis*-regulatory regions. We found two distal genomic regions that had enhancer activity in endothelial cells and in the pharyngeal epithelium and somites, respectively. The novel endothelial enhancer was conserved across species and was specific to large-caliber arteries. Its transcriptional activity was regulated by Notch signaling *in vitro* and *in vivo*, but not by ALK1 signaling and other transcription factors implicated in endothelial cell specificity. The distal endothelial enhancer was not essential for basal *Hey1* expression in mouse embryos but may likely serve for Notch-dependent transcriptional control in endothelial cells together with the proximal regulatory region. These findings help in understanding the significance and regulation of endothelial *Hey1* as a mediator of multiple signaling pathways in embryonic vascular formation.

Transcription factors play essential roles in complex arrays of developmental events and are implicated in the etiologies of various human diseases (1, 2). Multiple upstream signals regulate their expression and function in a cell type– and stage–specific manner, which in turn deeply influences cellular differentiation, proliferation, and movement through transcriptional control of downstream target genes. We and others previously identified the Hey family of basic helix-loop-helix transcriptional repressors that were enriched in the embryonic cardiovascular system (3–8). Among three family members, the mice null for *Hey2* die soon after birth, showing cardiac malformations and abnormal chamber gene expression (9–12). Combined loss of *Hey1* and *Hey2* resulted in embryonic lethality due to impaired vascular network formation (11, 13, 14). In addition, we recently reported that the *Hey1* deficiency caused lethal anomalies of the thoracic great vessels (15), which were similar to human congenital defects observed as isolated cardiovascular anomalies or as a manifestation of multiorgan syndromes such as 22q11.2 deletion syndrome (16). *Hey1* null mice should serve as a new experimental model for human great vessel malformations, which possesses unique as well as overlapping features compared with existing mouse models (17–27). However, the relative importance of Hey1 actions in vascular cell types is not fully elucidated.

Among a variety of cellular signaling pathways involved in embryonic development, it was first demonstrated that Notch signal activation stimulated the expression of *Hey* family genes through the Rbpj-dependent transcriptional control (28, 29). *Hey1* appears sensitive to Notch signaling *in vivo*, and the deletion of *Rbpj* or the Notch ligand *Dll1* results in down-regulation of *Hey1* expression in mouse embryos (5, 30–32). In addition, bone morphogenetic protein (BMP)-ALK receptor signaling activates the *Hey1* and *Hey2* transcription, which acts synergistically with Notch signaling in endothelial cells (33–35). Members of the *Hey* genes are under the control of other signaling pathways, such as those mediated by transforming growth factor β, hepatocyte growth factor, fibroblast growth factor, and Wnt (36–39). Despite these lines of evidence, it is not clear how these upstream signaling pathways control cell type specificity of the *Hey* family expression in various embryonic tissues. For example, *Hey1* and *Hey2* are mutually exclusive in the atrial and ventricular myocardium, whereas they look co-regulated in the atrioventricular canal and endocardium (3, 4). Expression in the pharyngeal epithelium is observed only for *Hey1*, whereas that in the dorsal ganglion is specific to *Hey2*. Detailed analyses of transcriptional regulation are necessary to further dissect distinct and complementary roles of *Hey* family genes in different embryonic tissues.

In this study, we generated *Hey1* conditional knockout (cKO) mice and demonstrated that the *Hey1* expression in endothelial cells was indispensable for proper pharyngeal arch artery (PAA) development and great vessel morphogenesis. We further performed a bacterial artificial chromosome (BAC)-based enhancer screen and identified distal enhancers that were specific to different cell types with *Hey1* expression. The novel endothelial enhancer possesses transcriptional activity recapitulating endogenous endothelial expression in large-caliber arteries downstream of Notch signaling. These data will provide important information to further understand *Hey1* function and its regulatory mechanisms in cardiovascular development and disease.

## Results

### The Hey1 gene in endothelial cells is essential for thoracic great vessel development

**Figure 1.**
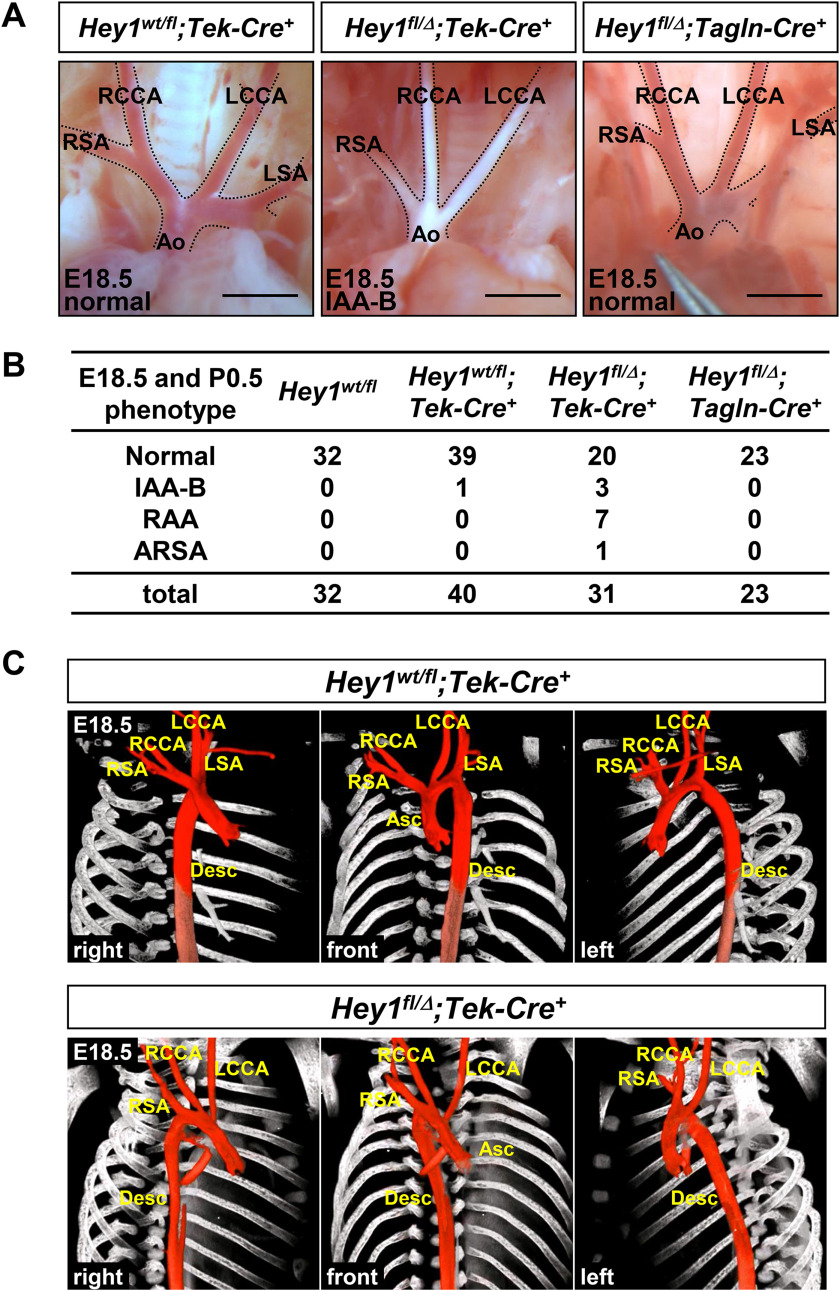
**Loss of endothelial *Hey1* leads to thoracic great vessel malformations.** *A*, *Hey1* endothelial cKO (*Hey1^fl/^*^Δ^*;Tek-Cre*^+^) mice showed IAA-B, whereas control (*Hey1^wt/fl^;Tek-Cre*^+^) and smooth muscle cKO (*Hey1^fl/^*^Δ^*;Tagln-Cre*^+^) mice had normal great vessel structures at E18.5. *Ao*, aorta; *RCCA/LCCA*, right/left common carotid artery; *RSA/LSA*, right/left subclavian artery. *Scale bars*, 1 mm. *B*, summary of great vessel phenotypes at E18.5 and postnatal day 0.5 (*P0.5*). *C*, front and right/left anterior oblique views of micro-CT images at E18.5. Right-sided aortic arch and unordinary branching were observed in an endothelial cKO (*Hey1^fl/^*^Δ^*;Tek-Cre*^+^) mouse. *Asc*, ascending aorta; *Desc*, descending aorta. Three-dimensional movies are available in the supporting information.

To clarify a cell type that requires *Hey1* function for the great vessel formation, we generated a novel *Hey1* cKO mouse line (Fig. S1). *Hey1* is expressed in both endothelial and smooth muscle cells in developing vasculature (14), so we ablated the *Hey1* gene in endothelial and smooth muscle cells using *Tek-Cre* and *Tagln-Cre* mice (40, 41), respectively. The *Tek-Cre*–mediated *Hey1* deletion in endothelial cells caused abnormalities of great vessel structure, namely interruption of the aortic arch type B (IAA-B), right-sided aortic arch (RAA), and aberrant origin of the right subclavian artery (ARSA) at embryonic day 18.5 (E18.5) and postnatal day 0.5 (Fig. 1, *A* and *B*), which reproduced congenital anomalies observed in *Hey1* null mice (15). We did not detect morphological defects of the heart and signs of circulation failure in *Tek-Cre*–mediated cKO embryos. Micro-CT analysis clearly showed the three-dimensional image of unordinary running and branching of the aorta in cKO embryos with RAA at E18.5 (Fig. 1*C* and Movies S1 and S2). On the other hand, the *Hey1* deletion in vascular smooth muscle cells did not affect great vessel morphogenesis (Fig. 1, *A* and *B*). These results indicated that endothelial *Hey1* expression was indispensable for the thoracic great vessel formation.

### Loss of endothelial Hey1 expression leads to disrupted tubular structure of fourth PAAs

**Figure 2.**
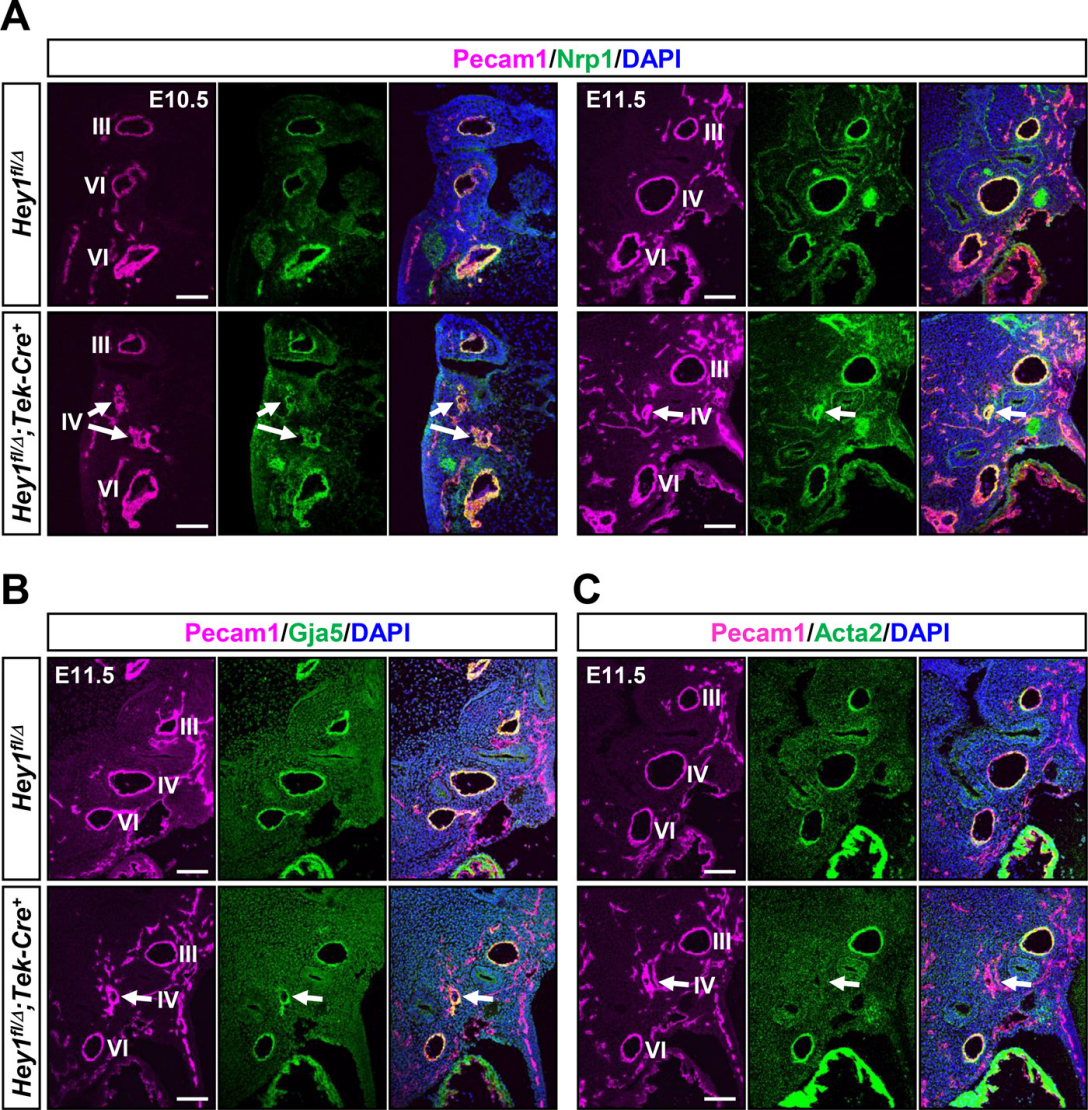
**Embryonic fourth PAA formation is disrupted by endothelial *Hey1* deficiency.** *A*, immunohistochemistry of Pecam1 and Nrp1 revealed defective tubular structure of fourth PAAs (*arrows*) in endothelial cKO (*Hey1^fl/^*^Δ^*;Tek-Cre*^+^) embryos at E10.5 and E11.5. *B*, Gja5 immunohistochemistry at E11.5 also showed the impaired endothelial tube formation of fourth PAAs. Expression levels of these endothelial markers remained unchanged. *C*, expression of a smooth muscle marker Acta2 was almost undetectable in the affected fourth PAA. PAAs are numbered. Note that the staining of different markers is shown using serial sections for E11.5. *Scale bars*, 100 μm.

IAA-B, RAA, and ARSA are all attributable to the fourth PAA defects at early developmental stages (42). We then analyzed the structures of pharyngeal arches and PAAs of endothelial cKO embryos. In control embryos, third, fourth, and sixth PAAs were well-formed in corresponding pharyngeal arches by E10.5 (Fig. 2*A*). In contrast, the disruption of endothelial tube structure was observed in fourth PAAs of endothelial cKO embryos at E10.5 and E11.5 (Fig. 2*A*), whereas the size and structure of pharyngeal arches were maintained. Pan-endothelial (Pecam1) and arterial endothelial (Nrp1 and Gja5) markers were clearly expressed in the disorganized vasculature (Fig. 2, *A* and *B*). Quantitative RT-PCR analysis of Pecam1^+^ endothelial cells indicated that other endothelial marker genes were also expressed at normal levels in *Hey1* cKO as well as null embryos (Fig. S2, *A* and *B*), suggesting that general endothelial cell differentiation was not compromised. Migration and distribution of neural crest–derived cells, which were marked with Tfap2a (AP2α) and Crabp1, were not altered by the *Hey1* deficiency (Fig. S3, *A* and *B*); on the other hand, the expression of a smooth muscle marker Acta2 (α smooth muscle actin) was almost undetectable in the affected fourth PAA (Fig. 2*C*). These characteristics were identical to that in *Hey1* null embryos (15), substantiating the importance of endothelial *Hey1* for fourth PAA-derived great vessel formation.

**Figure 3.**
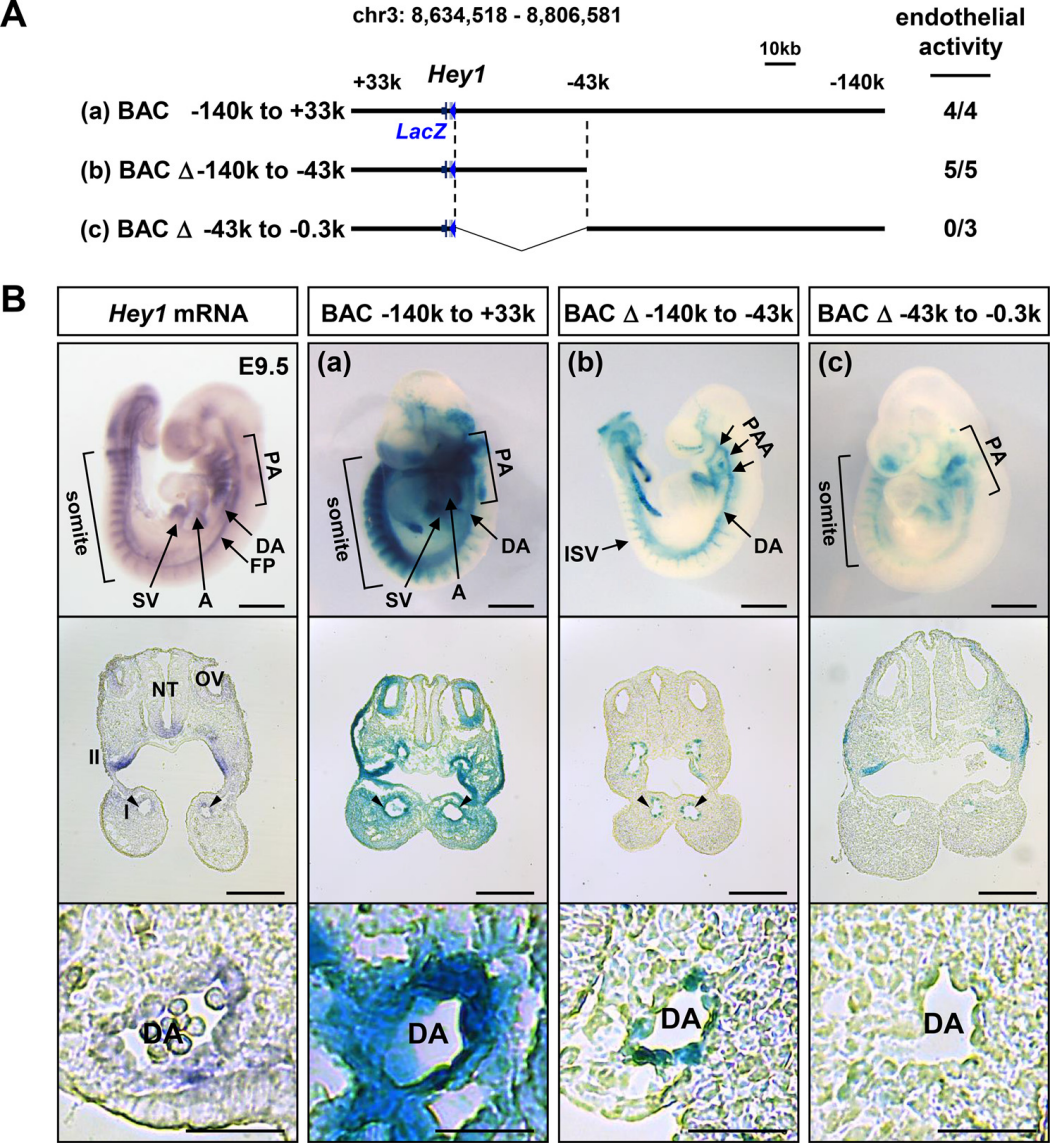
***Hey1* expression in embryonic tissues is regulated through multiple *cis*-regulatory regions.** *A*, a schematic of BAC-*LacZ* reporters for F0 transgenic mouse analyses is shown with the numbers of embryos with positive signals in vascular endothelial cells/embryos positive in any tissues. *B*, representative images of E9.5 transgenic embryos are shown with endogenous *Hey1* mRNA expression detected in *in situ* hybridization. β-Gal activity by the full-length BAC-*LacZ* reporter (*a*) reproduced endogenous *Hey1* mRNA expression in the dorsal aorta (*DA*), atrium (*A*), sinus venosus (*SV*), pharyngeal arch (*PA*), somites, and floor plate (*FP*) of neural tube (*NT*). Deletion of the −140 to −43 kb region resulted in the loss of activity in the atrium, pharyngeal epithelium, and somites (*b*), whereas that of the −43 to −0.3 kb region diminished the activity in DA, PAA, and intersomitic vessels (*ISV*) (*c*). Sections of whole-mount *in situ* hybridization are displayed to compare mRNA expression and reporter activity in PAAs. *Arrowheads*, PAAs with significant signals. *Magnified views* of DA are also shown at the *bottom*. PAAs are *numbered*. *OV*, otic vesicle. *Scale bars* in whole-mount images, sections, and magnified DA images are 500, 200, and 50 μm, respectively. Results of other F0 embryos are displayed in Fig. S5.

Great vessel anomalies occurred in some endothelial cKO mice but not in others (Fig. 1*B*), which was unrelated to the extent of *Hey1* down-regulation because its mRNA level in Pecam1^+^ cells did not markedly vary among cKO embryos (Fig. S2*A*). As is generally accepted for other genetic models showing great vessel malformations (24, 43–45), it is likely that endothelial *Hey1* deficiency predisposes cKO mice to fourth PAA defects. cKO embryos retained ∼30% of *Hey1* transcripts in the Pecam1^+^ endothelial cell population, whereas null embryos showed only negligible *Hey1* mRNA expression (Fig. S2*A*). As described in its original paper (40), the *Tek*-Cre-mediated recombination of the *Rosa* allele was observed in all identifiable endothelial cells by E10.5 (Fig. S4); however, the *Hey1* floxed allele should have relatively low efficiency of Cre-mediated recombination. There may be mosaicism of the *Hey1* allele recombination, although it is difficult to examine it with single-cell resolution. Nevertheless, it is of note that even a partial reduction of endothelial *Hey1* expression was enough to affect fourth PAA development.

**Figure 4.**
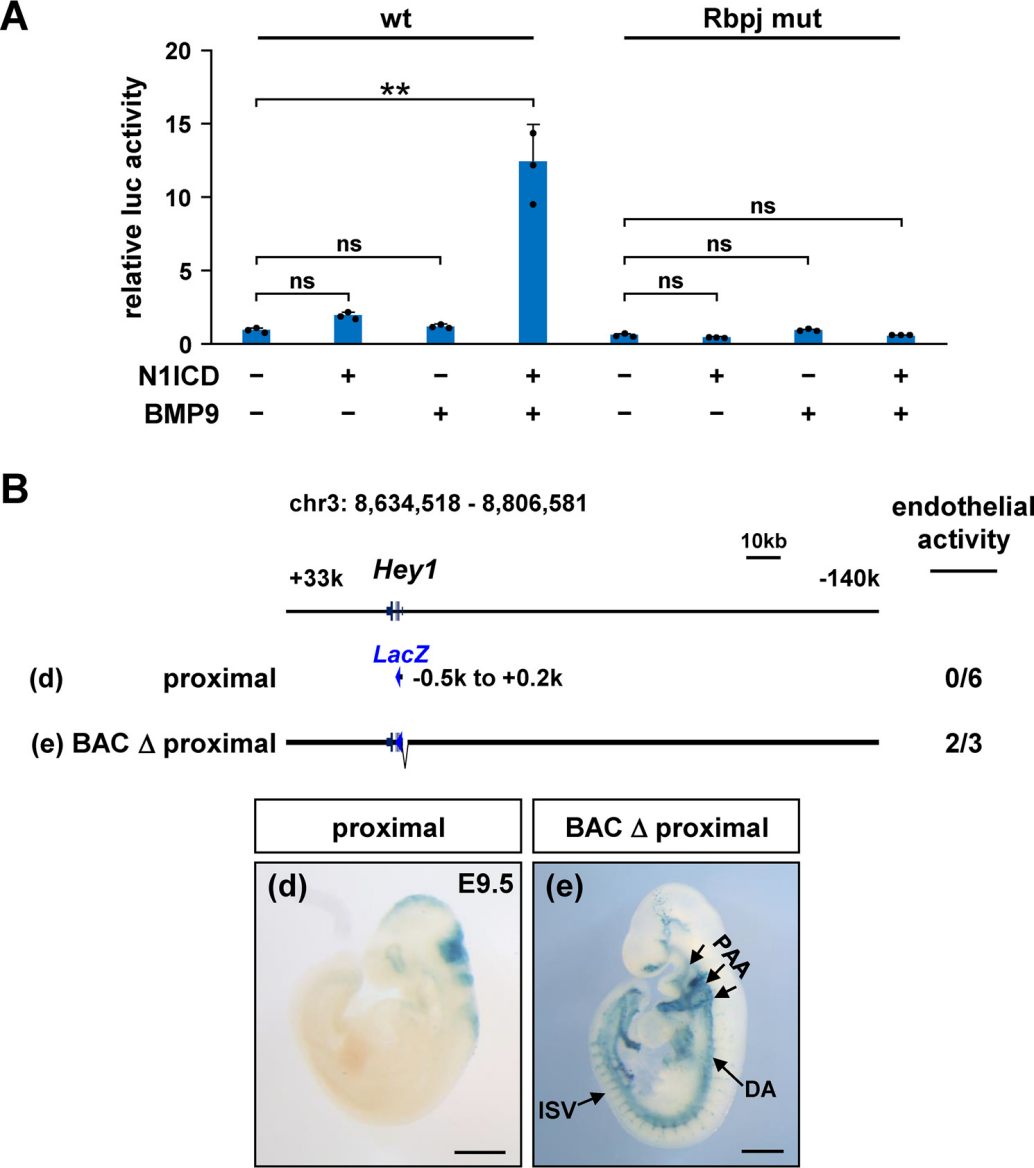
***Hey1* proximal region responds to vascular signals but is insufficient for endothelial transcription *in vivo*.** *A*, luciferase reporter assays in human umbilical vein endothelial cells revealed that the N1ICD expression and the BMP9 treatment synergistically induced the transcriptional activity driven by the proximal region, which required a Rbpj-binding site. The sequence of mouse *Hey1* proximal region is shown in Fig. S7. **, *p* < 0.01; *ns*, not significant. *B*, in F0 transgenic mouse reporter analyses, the proximal region was insufficient for endothelial transcription (*d*) and dispensable for the full-length BAC-*LacZ* reporter activity in vascular endothelial cells (*e*). *Scale bars*, 500 μm. Results of other F0 embryos are displayed in Fig. S7.

### Hey1 expression in embryonic tissues is regulated through multiple cis-regulatory regions

Considering the importance of endothelial *Hey1* expression for proper vascular development, we attempted identification of tissue-specific enhancers for *Hey1* transcription to understand how *Hey1* expression was regulated during embryonic development. The BAC construct that encompassed ∼173 kb surrounding mouse *Hey1* gene (−140 to +33kb) was used for F0 transgenic mouse *LacZ* reporter analysis (Fig. 3*A*). As shown in Fig. 3 (*A* and *B*) and Fig. S5 (*A* and *B*), the full-length BAC-*LacZ* reporter (designated as “a”) showed transcriptional activity that reproduced endogenous *Hey1* expression in the cardiovascular system, pharyngeal epithelium, and somites at E9.5. A shorter reporter that lacked the −140 to −43 kb region (b) lost the activity in the atria, pharyngeal epithelium, and somites but was positive specifically in large-caliber arteries, including PAA, the dorsal aorta, and intersomitic vessels. On the other hand, the deletion without the −43 to −0.3 kb region (c) did not drive the reporter expression in these arteries, whereas it retained the activity in the pharyngeal epithelium and somites. These results suggested presence of multiple *cis*-regulatory regions for *Hey1* transcription in different embryonic tissues.

**Figure 5.**
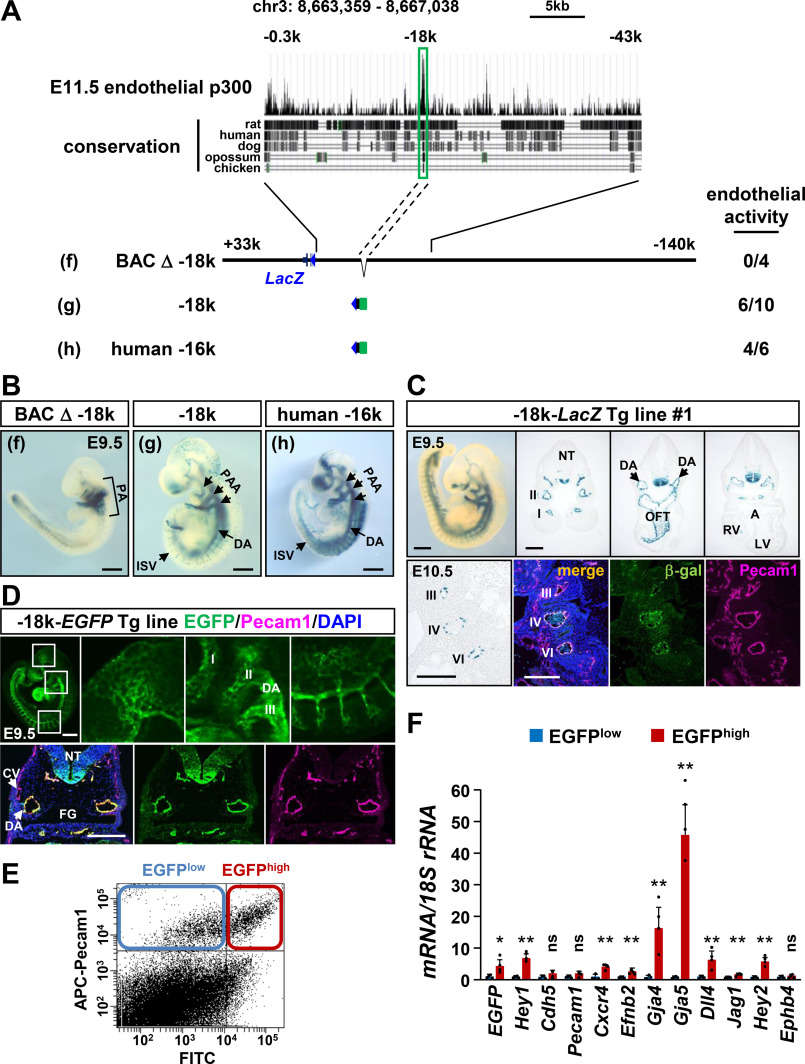
**A distal region of mouse *Hey1*/human *HEY1* functions as an endothelial enhancer.** *A*, the p300 ChIP-Seq result using E11.5 endothelial cells and genomic conservation proposed the mouse *Hey1* −18 kb region as an endothelial enhancer candidate. *B*, the BAC-*LacZ* reporter lacking the −18 kb region (*f*) did not induce robust β-gal activity in the vasculature of E9.5 embryos. Both the mouse −18 kb region (*g*) and the human −16 kb region (*h*) showed vascular activity in DA, PAAs, and ISV. Summary of the results is shown in *A*, and the results of other F0 embryos are displayed in Fig. S8. *C*, Tg mouse lines confirmed endothelial activity of the −18 kb-*LacZ* reporter in DA and PAAs (numbered). The floor plate of neural tube (*NT*) also expressed the *LacZ* reporter. *LV*, left ventricle; *OFT*, outflow tract; *RV*, right ventricle. *D*, the −18 kb-*EGFP* reporter Tg embryos recapitulated arterial endothelial expression of the *LacZ* reporter. *CV*, cardinal vein; *FG*, foregut. *Scale bars* in the whole-mount images and sections of *B*, *C*, and *D* are 500 and 200 μm, respectively. *E*, EGFP^high^ and EGFP^low^ populations of Pecam1^high^ endothelial cells were sorted from E10.5 embryos. *F*, real-time PCR analysis revealed that EGFP^high^ cells had high expression of *Hey1* and arterial markers (*Cxcr4*, *Efnb2*, *Gja4*, *Gja5*, *Dll4*, *Jagged1*, and *Hey2*), although the expression levels of pan-endothelial (*Cdh5* and *Pecam1*) and venous (*Ephb4*) genes were equivalent between EGFP^high^ and EGFP^low^ cells. *, *p* < 0.05; **, *p* < 0.01; *ns*, not significant.

The BAC-*LacZ* reporter analysis located the pharyngeal/somitic enhancer to the region between −140 and −43 kb. Consistently, a 0.6-kb fragment in the −50 kb region showed robust enhancer activity in the pharyngeal epithelium and somites (Fig. S6*A*). The activity of full-length BAC-*LacZ* reporter was reduced by the lack of the 0.6-kb fragment selectively in these tissues, whereas that in the vasculature was maintained (Fig. S6*A*). In addition, CRISPR/Cas9-mediated excision of the corresponding area significantly decreased endogenous *Hey1* expression in a tissue-specific manner (Fig. S6*B*), clearly indicating its sufficiency and necessity as a new pharyngeal/somitic enhancer.

**Figure 6.**
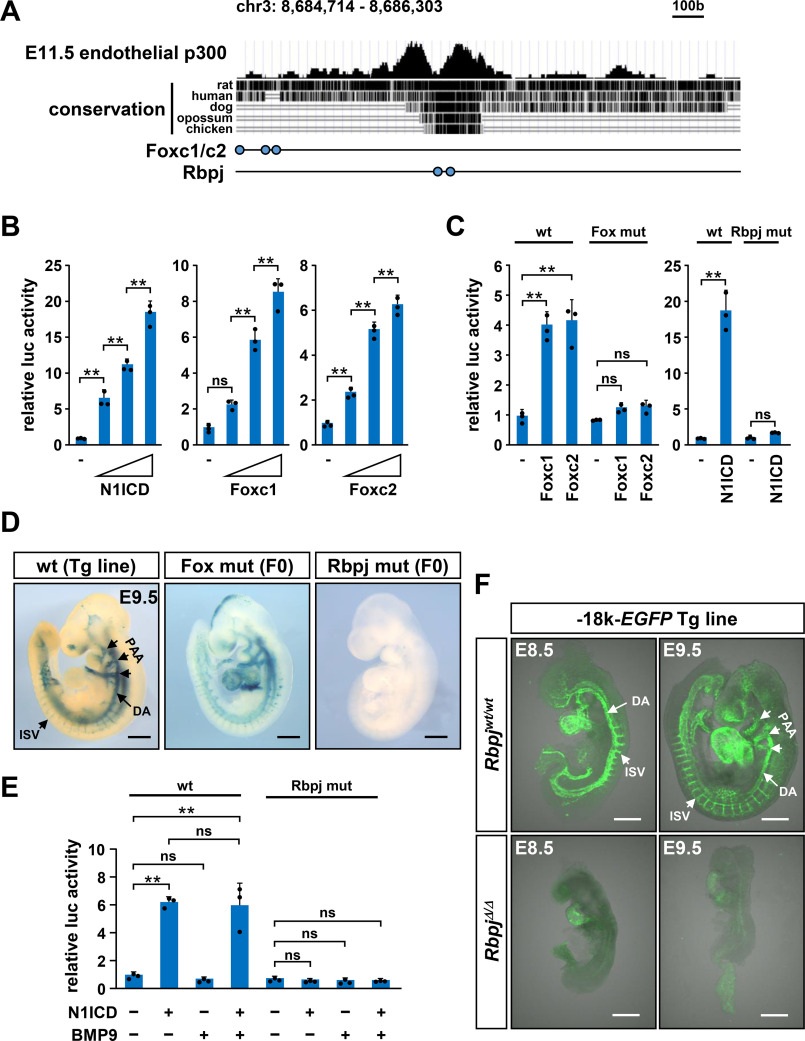
***Hey1* distal endothelial enhancer is controlled by Notch signaling.** *A*, the p300 ChIP-Seq result, genomic conservation, and binding sites for Foxc1/c2 and Rbpj at the −18 kb region of mouse *Hey1* gene are shown. *B* and *C*, expression of N1ICD or Foxc1/c2 dose-dependently induced the luciferase reporter activity driven by the −18 kb distal enhancer in 293T cells. Mutations in Rbpj- or Foxc-binding sites abolished the transcriptional activation and were used for *LacZ* reporter assays shown in *D*. **, *p* < 0.01; *ns*, not significant. *D*, the *LacZ* reporter activity driven by the −18 kb enhancer was lost by the mutations of Rbpj sites, but not by those of Foxc1/c2 sites, in E9.5 embryos. Results of other F0 embryos are shown in Figs. S10 and S11. *E*, in human umbilical vein endothelial cells, the N1ICD expression, but not the BMP9 treatment, induced the transcriptional activity of the −18 kb enhancer. Rbpj site mutations resulted in the loss of induction. *F*, endothelial *EGFP* expression by the −18 kb enhancer was almost undetectable in *Rbpj*^Δ/Δ^ mouse embryos at E8.5 and E9.5. *Scale bars* in *D* and *F*, 500 μm.

### Hey1-proximal region responds to vascular signals but is insufficient for endothelial specific transcription in vivo

BAC-*LacZ* studies indicated that the endothelial enhancer was present between −43 and −0.3 kb (Fig. 3, *A* and *B*), and we further examined how endothelial *Hey1* transcription was controlled in mouse embryos. It was previously reported that the *HEY1* expression was synergistically activated by Notch and ALK1 signaling in endothelial cells (Fig. S7*A*) (34). Transcriptional activity driven by the proximal region (−0.5 to +0.2 kb) was up-regulated by the Notch 1 intracellular domain (N1ICD) expression and BMP9 treatment (Fig. 4*A*) in luciferase reporter assays, which was consistent with the previous finding using BMP6 (33). The proximal region contained three potential Rbpj-binding sites, one of which was required for transcriptional activation (Fig. 4*A* and Fig. S7*B*).

Because Notch and ALK1 signaling pathways are heavily involved in endothelial differentiation and vascular formation (34, 35), we first tested the importance of the proximal region for endothelial specific transcription in mouse embryos. Unexpectedly, the proximal region did not show reproducible enhancer activity in the vasculature of *LacZ* reporter embryos (Fig. 4*B* and Fig. S7*C*). More importantly, the ablation of proximal region from the full-length BAC-*LacZ* reporter did not reduce the activity in vascular endothelial cells (Fig. 4*B* and Fig. S7*C*), strongly suggesting that the proximal region could serve for signal responsiveness but was insufficient to achieve endothelial transcription *in vivo*.

### A distal endothelial enhancer is located 18 kb upstream of the mouse Hey1 gene

With this result, it was the best conceivable that an additional enhancer for endothelial *Hey1* expression was present in the region between −43 and −0.3 kb. We then analyzed publicly available ChIP sequencing (ChIP-Seq) (Gene Expression Omnibus, under accession number GSE88789) data sets and found the transcriptional coactivator p300 binding 18 kb upstream of the mouse *Hey1* gene in embryonic endothelial cells (Fig. 5*A*) (46). Indeed, the ablation of a 1.6-kb sequence at the −18 kb region clearly deprived the full-length BAC-*LacZ* reporter of vascular transcriptional activity at E9.5 (Fig. 5 (*A* and *B*) and Fig. S8*A*). In addition, the *LacZ* reporter analysis indicated that the 1.6-kb fragment had highly specific enhancer activity in embryonic vasculature (Fig. 5 (*A* and *B*) and Fig. S8*A*). A detailed analysis using the reporter mouse lines revealed that the transcriptional activity was restricted to endothelial cells of large-caliber arteries, such as PAAs and the dorsal aorta at E9.5 and E10.5 (Fig. 5*C* and Fig. S8*B*).

The 1.6-kb sequence was highly conserved among species (Fig. 5*A*). A comparable 1.9-kb fragment in the human *HEY1* −16 kb region also showed *LacZ* reporter expression in vascular endothelial cells, suggesting relevance of the new, distal endothelial enhancer in human gene regulation (Fig. 5 (*A* and *B*) and Fig. S8*C*). To characterize the distal enhancer specificity, we further established an *EGFP* reporter line using the mouse −18 kb fragment. Similar to the *LacZ* reporter lines (Fig. 5*C*), the *EGFP* mice showed intense fluorescent signals in endothelial cells of large-caliber arteries, but not in the cardinal veins (Fig. 5*D*). Endogenous *Hey1* mRNA level was significantly higher in EGFP^high^ cells of the Pecam1^high^ endothelial population, and the expression of arterial endothelial genes was also enriched in EGFP^high^ endothelial cells (Fig. 5, *E* and *F*).

The newly identified distal enhancer drove endothelial transcription that recapitulated endogenous *Hey1* expression in large caliber arteries. Contrary to our expectation, however, CRISPR/Cas9-mediated excision of this region did not cause significant decrement of endogenous *Hey1* mRNA level in mouse embryos (Fig. S9, *A* and *B*). These results suggest that the proximal region or an unidentified enhancer may compensate the lack of the distal endothelial activity, whereas it was technically difficult to test such a hypothesis in mouse embryos by deleting multiple candidate regions around the *Hey1* gene.

### Hey1 distal endothelial enhancer is controlled by Notch signaling

Last, we examined regulatory mechanisms of the distal enhancer for its endothelial activity. In a screen for potential transcriptional regulators based on the consensus binding motif analysis, Foxc1, Foxc2, and N1ICD showed highest induction of the luciferase reporter expression driven by the distal enhancer (Fig. 6, *A* and *B*). The responsiveness to Foxc proteins was assigned to a 430-bp fragment (Fig. S10*A*) and three binding sites (Fig. 6, *A* and *C*). Nevertheless, their mutations in the 1.6-kb enhancer did not affect *LacZ* reporter expression in mouse embryos (Fig. 6*D* and Fig. S10*B*). In marked contrast, mutations in Rbpj-binding sites abolished the response to N1ICD in luciferase analysis (Fig. 6*C* and Fig. S11 (*A* and *B*)) and resulted in the complete loss of *LacZ* reporter activity in the embryonic vasculature (Fig. 6*D* and Fig. S11*C*). These Rbpj-binding elements are present in the corresponding genomic region of various species, suggesting that they are functional in humans and other creatures (Fig. S11*A*). There are no consensus motifs for SMAD binding in the distal enhancer, and ALK1 signaling did not show synergy with Notch signaling for the distal enhancer activation (Fig. 6*E*), unlike its effect for the proximal region (Fig. 4*A*).

As previously reported (31), *Hey1* mRNA expression in multiple embryonic tissues, including PAAs and the aorta, was markedly decreased in *Rbpj* null embryos, whereas severe developmental defects made its interpretation difficult (data not shown). We then analyzed whether the distal endothelial enhancer activity was dysregulated in *Rbpj* null embryos by intercrossing them with *EGFP* reporter mice. As shown in Fig. 6*F*, vascular EGFP expression was suppressed to a virtually neglectable level with the *Rbpj* null background even in E8.5 embryos showing relatively mild developmental defects. These results verified complete Notch dependence of the distal endothelial enhancer and further indicated its usefulness as a surrogate to monitor the Notch signal activity in the embryonic vasculature.

## Discussion

In this study, we demonstrate that the conditional deletion of *Hey1* in endothelial cells causes abnormalities of thoracic great vessel morphogenesis. Embryonic arterial defects occur only in fourth PAAs, which is identical to those observed in *Hey1* null mice (15). Fourth PAAs are prone to be disorganized during remodeling due to unique characteristics, including a nonmuscular region where the expression of α smooth muscle actin is reduced or absent (22, 47). Left and right fourth PAAs normally form a part of the aortic arch and right subclavian artery, and their defects cause IAA-B and ARSA, respectively. RAA results from the connection of right fourth PAA to the aorta, which compensates for the abnormal involution of left fourth PAA. It is still unclear how the *Hey1* deficiency in endothelial cells acts as a predisposing factor for such abnormalities. Among numerous signaling factors implicated in fourth PAA development (17, 19–21, 23, 24, 27, 48–51), *Hey1* may be functionally interconnected with endothelial regulatory genes, such as *Plxnd1* and *Edn1.* We performed RNA-Seq analysis of embryonic endothelial cells from *Hey1* endothelial cKO as well as null mice, but the results did not show the disturbance of a single gene or a few genes that explained vascular abnormalities by the *Hey1* deficiency. There was no significant enrichment that could be directly linked to the pathogenesis in gene ontology or KEGG pathway analysis (data not shown). ChIP-grade antibodies against Hey1 are not available, but ChIP-Seq was previously performed using FLAG-tagged HEY1 overexpression in cultured cells (52, 53). These studies suggested possible Hey1 target genes, including *Dll4*, *Kdr*, and *Foxc1*, but the expression of those candidate genes did not change in endothelial cells of *Hey1* cKO and null embryos (data not shown). Mosaicism of the *Hey1* allele recombination may have made such expression analyses difficult, and a specific population of endothelial cells in a particular region of the embryos might be more sensitive to the *Hey1* dosage. Importance in endothelial cells is also evident for complementary functions by *Hey1* and *Hey2* at earlier vascular formation because their loss in endothelial cells reproduces vascular defects and embryonic lethality observed in double-null mice (14). Advanced technologies such as multiomics analyses at the single-cell level may give a clue to downstream signaling pathways that are central to Hey-dependent endothelial functions.

Whereas the *Hey1* deletion in smooth muscle cells did not cause great vessel anomalies, that does not necessarily negate its supportive functions in smooth muscle cells. Consistently, the incidence of great vessel anomalies appeared lower in endothelial *Hey1* cKO mice compared with null mice (15). Although significant retention of *Hey1* transcripts in endothelial cells, probably due to low recombination efficiency of the *Hey1* floxed allele, can explain low phenotypic penetrance in cKO mice, Hey1 functions in smooth muscle cells or other cell types may also be required for proper fourth PAA development. However, it is noteworthy that *Hey1*-deficient phenotypes are clearly distinguishable from neural crest–related abnormalities. IAA-B and other great vessel malformations are often observed in patients with 22q11.2 deletion syndrome, and *TBX1* is one of the most important genes in the minideletion region (19, 54–56). Tbx1 transcription factor is required for migration, proliferation, and/or survival of neural crest–derived cells, and its mutant mice are defective also in the outflow tract, thymus, and craniofacial structures (19, 44, 55–57). In sharp contrast, *Hey1* null (15) as well as endothelial cKO mice (Fig. S3) did not show abnormalities in neural crest–derived cell behavior as well as malformation of the pharyngeal arches, cardiac outflow tract, and other neural crest–related structures. Mouse embryos with inactivation of Notch signaling in neural crest–derived cells showed down-regulation of *Hey* family expression in the smooth muscle layer of PAAs, but the fourth PAA defects only rarely occurred in these mutant mice (58). Furthermore, the heterozygous *Tbx1* deletion in the pharyngeal epithelium leads to great vessel abnormalities (59), indicating that Tbx1 and Hey1 control distinct signaling pathways for PAA development in different cell types.

The present study further elucidates that *Hey1* transcription in embryonic tissues is controlled through multiple *cis*-regulatory regions. In particular, endothelial *Hey1* expression essential for vascular development appears maintained through at least two complementary regions. The proximal region adjacent to exon 1 responds to both Notch and ALK1 signaling but is not sufficient to implement endothelial transcription in mouse embryos. The novel enhancer at the distal −18 kb region is solely regulated by Notch signaling and can reproduce the endogenous *Hey1* expression pattern in endothelial cells. Nonetheless, the distal enhancer does not single-handedly control endothelial *Hey1* transcription because its deletion does not alter *Hey1* mRNA level in embryonic endothelial cells. *In silico* analysis of ChIP-Seq data (46) indicates a couple of additional p300-binding sites around the *Hey1* locus (data not shown), although they are located outside the minimal −43 to −0.4 kb region determined in BAC-*LacZ* analysis. As is often seen with essential regulatory genes for embryonic development (60, 61), it is likely that the proximal, distal, and possibly additional regulatory regions supply the place of each other to ensure robust *Hey1* expression in endothelial cells.

*Hey1* and other *Hey* family genes have been recognized as typical Notch downstream genes enriched in the cardiovascular system (3, 28), and both the *Hey1* proximal region and distal endothelial enhancer have Rbpj-binding elements that mediate Notch responsiveness. Notch signaling is implicated in early steps of endothelial differentiation, such as arterial-venous specification and tip cell–stalk cell interaction (62, 63). In addition, fluid shear stress activates Notch signaling and up-regulates the expression of Notch target genes, including *Hey1* (64). It is known that an adequate range of blood flow into developing PAAs is necessary for proper morphogenesis of great vessel structure (65), and Notch-dependent *Hey1* expression is probably an important factor that responds to the variable blood flow during drastic PAA remodeling. The present study suggests that a decrement of *Hey1* expression in endothelial cells can predispose embryos to PAA defects. Strict regulation of *Hey1* expression is likely crucial to prevent congenital malformations of thoracic great vessels.

The *Hey1* distal enhancer activity in arterial endothelial cells is abolished by the mutations of Rbpj-binding sites and is clearly repressed in *Rbpj* null embryos. Other arterial endothelium enhancers show different modes of Rbpj dependence. The *Dll4* enhancer is regulated by Notch signaling in combination with SoxF and Ets factors, but its activity is influenced neither by the Rbpj site mutations nor by *Rbpj* deficiency (66, 67). The *Notch1* and *Ece1* endothelial enhancers do not contain Rbpj sites but display arterial specificity by the actions of SoxF, Ets, and Foxc factors (68, 69). On the other hand, Rbpj acts as a transcriptional repressor in venous endothelial cells for the *Flk1* arterial enhancer (70). Such a diversity of regulatory mechanisms is certainly important for unique characteristics of their expression.

During embryonic development, *Hey1* is expressed in various other tissues, such as the pharyngeal epithelium, somites, and cardiac atrium (3, 4). This study further identified a distal enhancer for the pharyngeal epithelium and somites. Marked reduction of endogenous *Hey1* expression by its deletion clearly shows importance as a specific enhancer, but a low level of *Hey1* mRNA is still detectable in these tissues. Because the BAC-*LacZ* reporter activity in these two areas is suppressed by the lack of proximal region (Fig. 4*B*), the proximal region may serve for *Hey1* transcription also in the pharyngeal epithelium and somites. In addition, *Hey1* expression restricted to the atrium of the heart is particularly interesting because its family gene *Hey2* is differentially expressed in the ventricle (3, 4). We recently reported that ventricular *Hey2* expression was controlled by Tbx20 and Gata family proteins through its distal enhancer (71). Although the full-length BAC apparently contains transcriptional activity in the atrium, we have not specified where the *Hey1* atrial enhancer is located. Studying the precise mechanisms of *Hey1* transcriptional regulation will help understand how tissue-specific gene expression is achieved in the cardiovascular system and other organs during embryonic development.

Increasing evidence supports the importance of the *HEY* family in human physiology and disease. Their expression is often dysregulated downstream of Notch signaling in various diseases (72–74). In addition, a fusion of *HEY1* and *NCOA1* causes chondrosarcoma and aberrant *HEY1* expression is correlated with the metastasis, therapeutic response, and patient survival in multiple cancer types (38, 75–78). A variant near the *HEY2* locus is associated with clinical characteristics of Brugada syndrome (79), and HEY2 also acts as a key factor for the differentiation of pluripotent stem cells into ventricular myocytes (80). It is tempting to hypothesize that variations in the protein-coding or enhancer regions of the *HEY1* gene affect its molecular function or expression level in human patients with isolated great vessel anomalies. *HEY1* may also be associated with clinical variability of 22q11.2 deletion syndrome as a modifier gene. It will be of clinical interest to examine possible involvement of *HEY1* in such entities of human congenital diseases.

## Experimental procedures

### Mouse strains

The founder mice with the *Hey1* floxed allele were generated as follows (Fig. S1). The targeting vector was designed to delete exons 2, 3, and 4 of the *Hey1* gene by Cre-mediated recombination and was electroporated into HK3i ES cells (81) with two single guide RNAs (Table S1) and the human optimized Cas9 expression plasmid px330 (Addgene plasmid 42230) (82). Recombinant ES cells were injected into eight-cell stage embryos to produce chimera mice. The Pgk-Neo cassette flanked by FRTs was removed by crossing the chimera mice with Flp-expressing female mice (Jackson Laboratory, JAX 003800). PCR primers for the genotyping are shown in Fig. S1 and Table S1. The *Hey1(loxP)* line information is available at RRID:SCR_019138 (accession no. CDB1302K). *Tek-Cre* (JAX 008863) and *Tagln* (*SM22*α)*-Cre* (JAX 004746) mice were used for the tissue-specific deletion (40, 41).

The mouse lines with the enhancer deletion at the *Hey1* −18 or −50 kb region were generated by CRISPR/Cas9 genome editing. Human optimized *Cas9* mRNA or Alt-R S. p. Cas9 Nuclease 3NLS (Integrated DNA) was introduced into BDF1 fertilized eggs with two single guide RNAs (Table S1) (83). The targeted regions were PCR-amplified for the sequencing at the F0 and F1 generations. Mice at the F1 and later generations were used for expression and phenotype analyses.

All animal experiments were approved by the institutional animal care and use committees of the National Cerebral and Cardiovascular Center and RIKEN Kobe Branch.

### Micro-CT imaging

A contrast reagent eXIA 160XL (Summit Pharmaceutical International) was introduced from the right ventricle of E18.5 embryos, and micro-CT images were taken with an 8-μm pixel size using SkyScan 1276 (Bruker).

### Fluorescence and magnetic activated cell sorting (FACS and MACS)

E10.5 mouse embryos were minced and incubated in 0.1% collagenase type II (Worthington, catalog no. 4176) with mixing and suspension. The dissociated cells were filtered through a BD Falcon Cell Strainer (70 μm, catalog no. 352350) and resuspended in 0.1% BSA and 2 mm EDTA in Ca^2+^/Mg^2+^-free PBS. For FACS, the cells were incubated with phycoerythrin– or APC-Cy7–conjugated α-mouse CD31 (Pecam1) antibody MEC13.3 (BioLegend, catalog no. 102507 or 561814) and FITC-conjugated α**-**mouse CD45 antibody 30-F11 (BioLegend, catalog no. 103107), followed by sorting with FACS Aria (BD Biosciences). For MACS, the cells were incubated with CD31 microbeads (Miltenyi Biotec, catalog no. 130-097-418) and sorted using MS columns (Miltenyi Biotec, catalog no. 130-042-201).

### Expression analysis

Total RNA was extracted from the FACS- or MACS-sorted cells using NucleoSpin RNA XS (Macherey Nagel), and cDNA was synthesized using the PrimeScript RT reagent kit (Takara). Real-time PCR analysis was performed with KAPA SYBR FAST qPCR Master Mix (Kapa Biosystems) and a LightCycler 96 system (Roche Applied Science). 18S rRNA was used as a reference. Primer sequences are listed in Table S1. The following antibodies were used for immunohistochemistry on frozen sections: α-Pecam1 (MEC13.3, BD Pharmingen, catalog no. 550274), phycoerythrin-conjugated α-Pecam1 (MEC13.3, BioLegend, catalog no. 102507), α-Nrp1 (Abcam, catalog no. ab81321), α-Gja5 (Invitrogen, catalog no. 36-4900), Cy3-conjugated α-Acta2 (Sigma, catalog no. 6198), and α-Tfap2a (Developmental Studies Hybridoma Bank, catalog no. 3B5). Whole-mount *in situ* hybridization was performed as we described previously (71). Statistical analysis was performed using Student's *t* test.

### LacZ reporter analysis

A BAC clone encompassing the mouse *Hey1* gene, *RP23-255P16*, was purchased from Advanced GenoTechs Co. The BAC-*LacZ* reporter construct was generated by inserting the nuclear localization signal (nls)-*LacZ*-poly(A) fragment at the first ATG site of the *Hey1* gene as we reported previously (84). Deletion BAC series were prepared with the *kanamycin^R^* insertion. Plasmid-based *LacZ* reporters of the proximal region and distal enhancers were made using the nls-*LacZ*-poly(A) and *hsp68* promoter-nls-*LacZ*-poly(A) vectors, respectively. Rbpj-binding sites in the −18 kb enhancer were mutated as follows: site 1, ACGTGATGGGAATTGGA → ACGTGActttAATTGGA; site 2, GGAGCGTGGGAACCCCG → GGAGCGctttAACCCCG. Foxc-binding sites were mutated as follows: site 1, AGCTTTATTGAGATACA → AGCTTaAagGAGATACA; site 2, TCACTTAAAATATGTGA → TCACTTAActTtTGTGA; site 3, TAGAATATTTTCATTTT → TAGAAaAagTTCATTTT. Transgenic mice were generated using standard methods (71). In brief, the circular BAC reporter or linear enhancer reporter fragment (1–3 ng/μl) was injected into the pronuclei of BDF1 fertilized eggs. β-Gal reaction of mouse embryos was performed as we described previously (71).

### Luciferase reporter analysis

A DNA fragment of the −18 kb enhancer was inserted into the pGL4.10[luc2] plasmid containing the MLP promoter (85). Human umbilical vein endothelial cells were seeded in the EGM2 medium (Lonza) 24 h before the transfection, and the plasmids for the luciferase, CMV-β-gal, and mouse N1ICD expression were introduced using Lipofectamine PLUS and LTX reagents (Life Technologies, Inc.). After 1 h of transfection, the cells were preconditioned in the EBM2 medium (Lonza) containing 0.2% BSA and 0.2% fetal bovine serum for 4 h, followed by the treatment with BMP9 (R&D Systems) or vehicle. Transfection of 293T cells was performed according to our previous report (86). Luciferase and β-gal activities were measured using FLUOStar Omega (BMG LABTECH). Triplicated assays were independently performed three times, which gave reproducible results. Statistical analysis was performed using Tukey's test.

## Data availability

All data are contained within the article and supporting information.

## Supplementary Material

Supporting Information
