# Supplementary material for: Importance of endothelial Hey1 expression for thoracic great vessel development and its distal enhancer for Notch-dependent endothelial transcription
Source: J Biol Chem. 2020 Oct 16;295(51):17632–45. doi: 10.1074/jbc.RA120.015003 (PMC7762959; doi:10.1074/jbc.RA120.015003)
Supplement: Supporting Information [file supp_295_51_17632__index.html]

Importance of endothelial Hey1 expression for thoracic great vessel development and its distal enhancer for Notch-dependent endothelial transcription — Endothelial Hey1: great vessel development and enhancer — Importance of endothelial Hey1 expression for thoracic great vessel development and its distal enhancer for Notch-dependent endothelial transcription — Endothelial Hey1: Great vessel development and enhancer — Supporting Information 

# Importance of endothelial *Hey1* expression for thoracic great vessel development and its distal enhancer for Notch-dependent endothelial transcription

## Supporting Information

- Supporting Information (to be published online) - Supporting information
- Movie S1 - Movie S1
- Movie S2 - Movie S2
