# Supplementary material for: Importance of endothelial Hey1 expression for thoracic great vessel development and its distal enhancer for Notch-dependent endothelial transcription
Source: J Biol Chem. 2020 Oct 16;295(51):17632–45. doi: 10.1074/jbc.RA120.015003 (PMC7762959; doi:10.1074/jbc.RA120.015003)
Supplement: Supporting Information [file supp_RA120.015003_161805_2_supp_610718_qd2q40.pdf]

Supporting information for:

Importance of endothelial *Hey1* expression for thoracic great vessel development and its distal enhancer for Notch-dependent endothelial transcription

Yusuke Watanabe[1,2,*], Daiki Seya[1], Dai Ihara[1,3], Shuhei Ishii[1,2], Taiki Uemoto[1,2], Atsushi Kubo[4], Yuji Arai[1,5], Yoshie Isomoto[5], Atsushi Nakano[5], Takaya Abe[6], Mayo Shigeta[6], Teruhisa Kawamura[3], Yoshihiko Saito[2,7], Toshihiko Ogura[4], Osamu Nakagawa[1,2,*]

[1] Department of Molecular Physiology, National Cerebral and Cardiovascular Center Research Institute, 6-1 Kishibe-shimmachi, Suita, Osaka, 564-8565, Japan
[2] Graduate School of Medical Sciences, Nara Medical University, Kashihara, Nara, 634-8521, Japan
[3] Laboratory of Stem Cell & Regenerative Medicine, Department of Biomedical Sciences, College of Life Sciences, Ritsumeikan University, 1-1-1 Noji-Higashi, Kusatsu, Shiga, 525-8577, Japan
[4] Department of Developmental Neurobiology, Institute of Development, Aging and Cancer, Tohoku University, 4-1 Seiryo-cho, Aoba, Sendai, Miyagi 980-8575, Japan
[5] Laboratory of Animal Experiment and Medical Management, National Cerebral and Cardiovascular Center Research Institute, 6-1 Kishibe-shimmachi, Suita, Osaka, 564-8565, Japan
[6] Laboratory for Animal Resources and Genetic Engineering, RIKEN Center for Biosystems Dynamics Research, 2-2-3 Minatojima Minami-machi, Chuou-ku, Kobe 650-0047, Japan
[7] Department of Cardiovascular Medicine, Nara Medical University, Kashihara, Nara, 634-8522, Japan

*Corresponding author
Yusuke Watanabe, Ph.D.
e-mail address: ywatanabe@ncvc.go.jp
Osamu Nakagawa, M.D., Ph.D.
e-mail address: osamu.nakagawa@ncvc.go.jp

Running title: Endothelial *Hey1*: great vessel development and enhancer

Keyword: Hey1, pharyngeal arch artery, great vessel morphogenesis, endothelial cell, transcriptional regulation, Notch signaling

Supporting information includes:

Supplemental Experimental Procedures
Figures S1 to S11
Table S1
Movies S1 and S2

Supplemental experimental procedures

Dual detection of the β-galactosidase activity and Pecam1 expression

E10.5 embryos obtained from *Rosa26R-LacZ* mice (1) crossed with *Tek-Cre* (2) mice were fixed in 4% paraformaldehyde for 30 min. Dual detection of β-galactosidase activity and Pecam1 expression was performed using the SPiDER-β-gal system (Dojindo) and α-Pecam1 antibody (BD Pharmingen #550274), as we previously described (3). The fluorescent images were taken using FV3000 confocal microscope (Olympus).

Electrophoretic mobility shift assay

Protein lysates were prepared from 293T cells transfected with the FLAG-tagged mouse Rbpj expression plasmid using the lysis buffer (10mM Tris-Cl pH7.8, 60mM KCl, 1mM EDTA, 0.1% NP40 and cOmplete EDTA-free Protease Inhibitor Cocktail (Roche #11873580001), followed by nuclear protein extraction using the buffer containing 20mM Tris-Cl pH7.8, 420mM NaCl, 1.5mM $MgCl_2$, 0.2mM EDTA, 25% glycerol and protease inhibitors. Oligonucleotide labelling with digoxigenin-11-ddUTP (Roche) and electrophoresis were performed as we previously described (4).

References

1. Soriano, P. (1999) Generalized lacZ expression with the ROSA26 Cre reporter strain. *Nat Genet* **21**, 70-71
2. Kisanuki, Y. Y., Hammer, R. E., Miyazaki, J., Williams, S. C., Richardson, J. A., and Yanagisawa, M. (2001) Tie2-Cre transgenic mice: a new model for endothelial cell-lineage analysis in vivo. *Dev Biol* **230**, 230-242
3. Ihara, D., Watanabe, Y., Seya, D., Arai, Y., Isomoto, Y., Nakano, A., Kubo, A., Ogura, T., Kawamura, T., and Nakagawa, O. (2020) Expression of Hey2 transcription factor in the early embryonic ventricles is controlled through a distal enhancer by Tbx20 and Gata transcription factors. *Dev Biol* **461**, 124-131
4. Watanabe, Y., Zaffran, S., Kuroiwa, A., Higuchi, H., Ogura, T., Harvey, R. P., Kelly, R. G., and Buckingham, M. (2012) Fibroblast growth factor 10 gene regulation in the second heart field by Tbx1, Nkx2-5, and Islet1 reveals a genetic switch for down-regulation in the myocardium. *Proc Natl Acad Sci U S A* **109**, 18273-18280

Figure S1

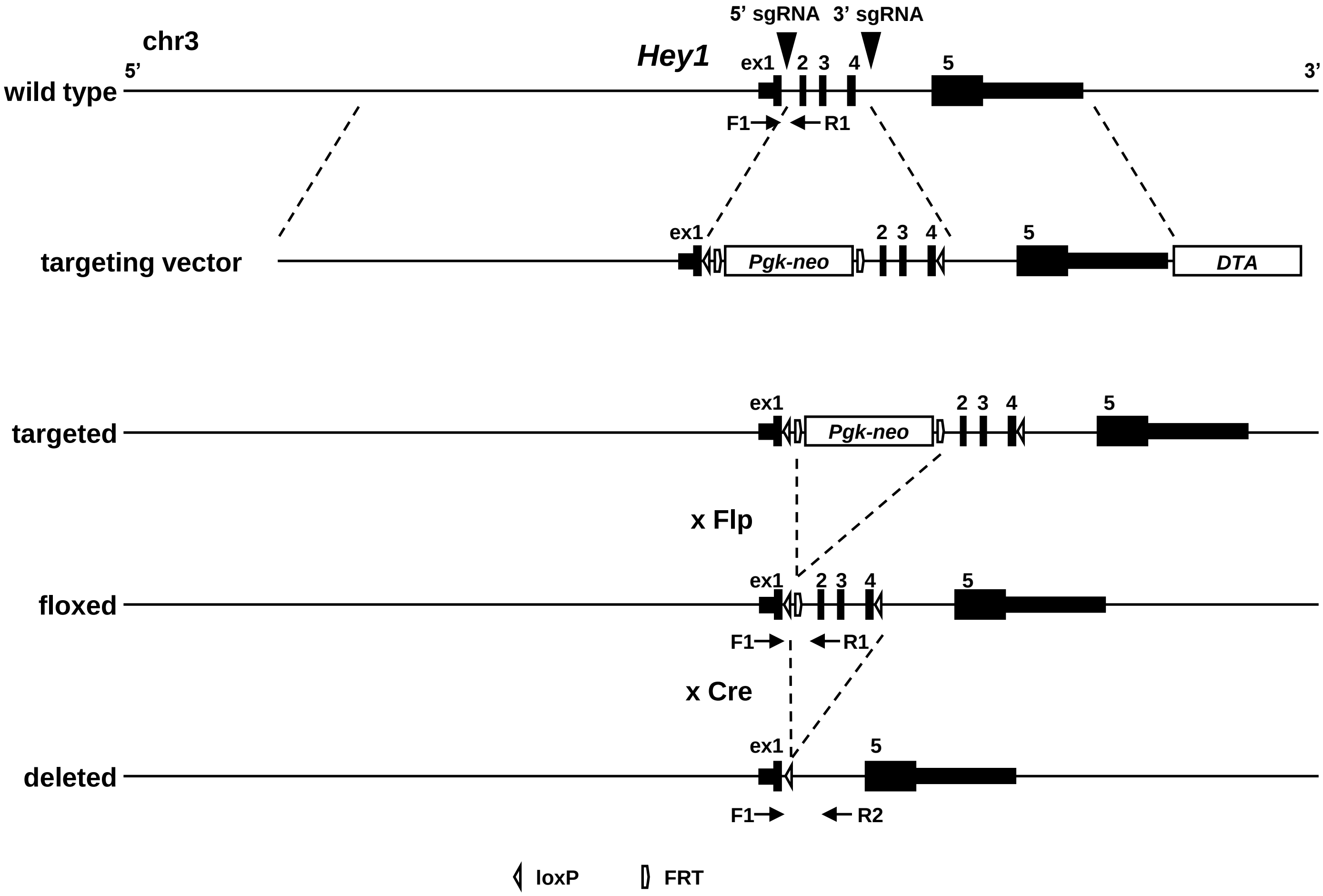

Fig. S1. A floxed allele of the *Hey1* gene was generated by CRISPR/Cas9-based homologous recombination using two single guide RNAs (sgRNAs) targeting intron 1 and 4. A *Pgk-neo* selection cassette was removed by the Flp recombinase. Exons 2 to 4 to be deleted by Cre recombinase encode the entire basic loop-helix-loop domain. Positions of genotyping primers are shown.

Figure S2

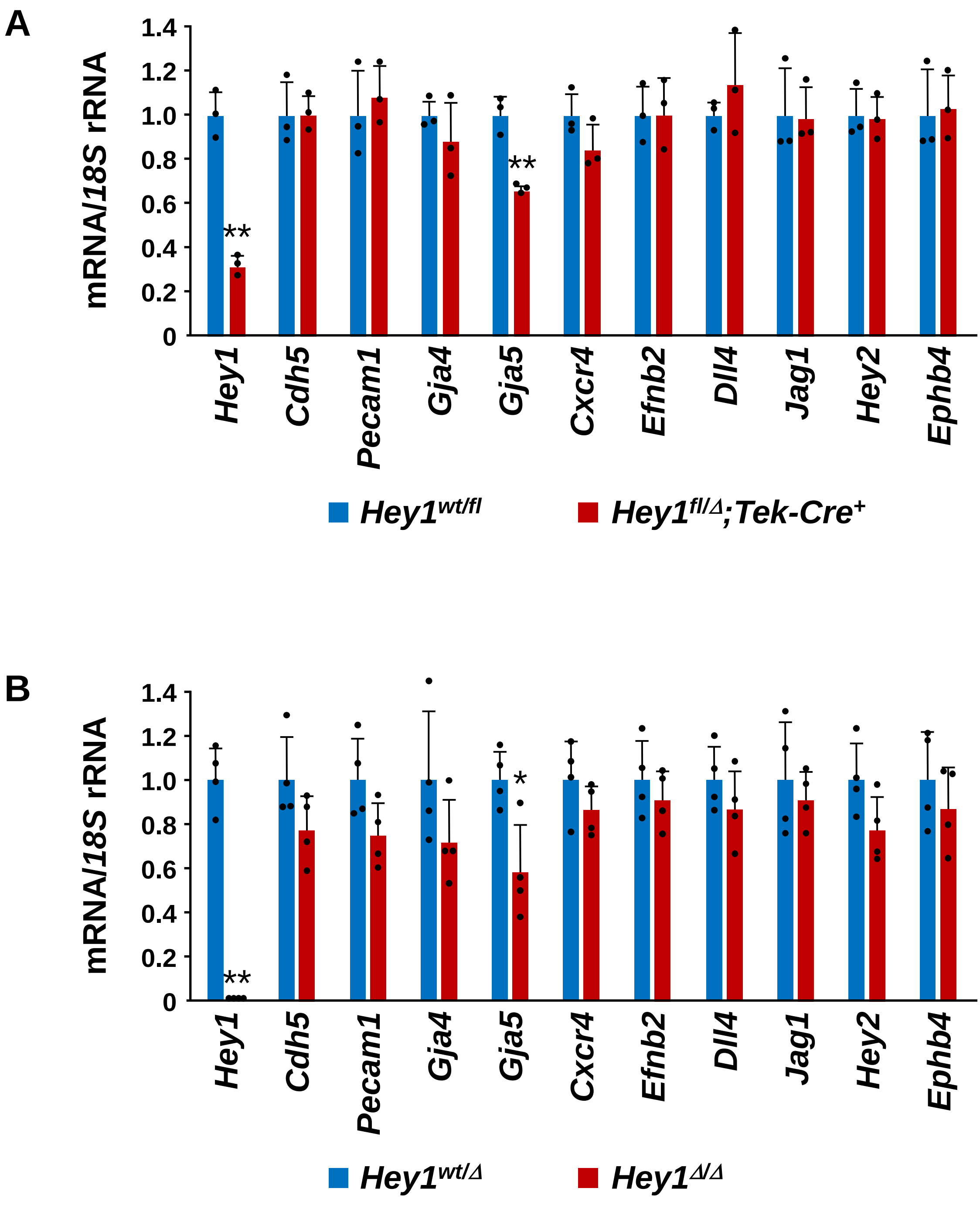

Fig. S2. Realtime PCR analysis was performed using Pecam1^{+} cells of control (*Hey1*$^{wt/fl}$) and *Hey1* endothelial cKO (*Hey1*$^{fl/\Delta}$*;Tek-Cre*$^{+}$) embryos (A), or control (*Hey1*$^{wt/\Delta}$) and *Hey1* null (*Hey1*$^{\Delta/\Delta}$) embryos (B), at E10.5. Following endothelial marker gene expression was examined: pan-endothelium (*Cdh5* and *Pecam1*), artery (*Gja4*, *Gja5*, *Cxcr4*, *Efnb2*, *Dll4*, *Jag1* and *Hey2*) and vein (*Ephb4*). *, $P < 0.05$ and **, $P < 0.01$.

Figure S3

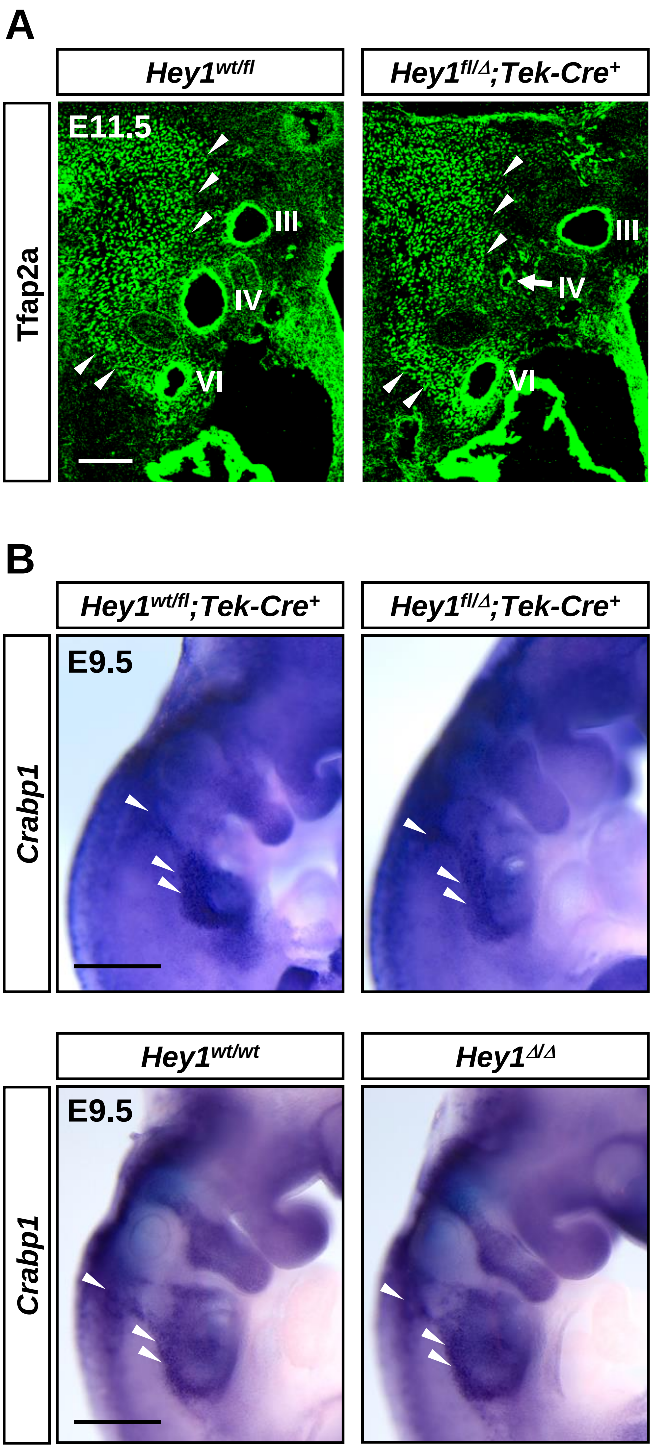

Fig. S3. Neural crest-derived cells were stained with Tfap2a/Ap2α (A) and *Crabp1* (B). Distribution of neural crest-derived cells was not affected in *Hey1* endothelial cKO (*Hey1*$^{fl/\Delta}$;*Tek-Cre*$^{+}$) and null embryos. White arrows indicate neural crest-derived cells in posterior pharyngeal arches. Scale bars are 100μm in a section and 500μm in whole mount views.

Figure S4

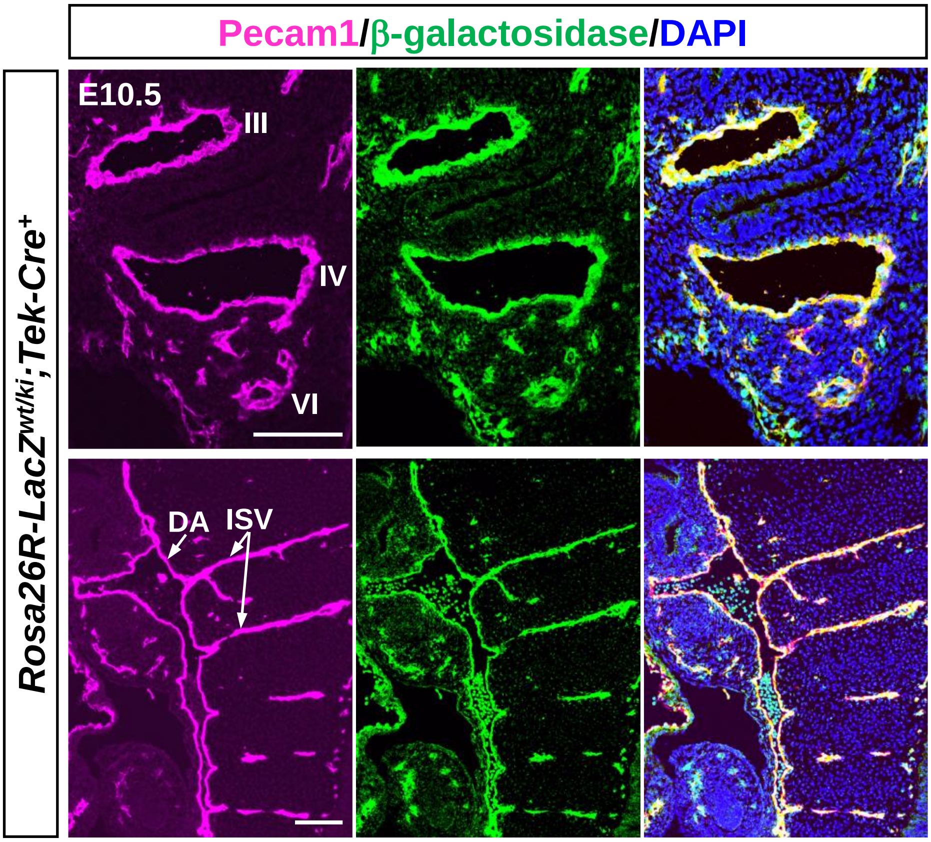

Fig. S4. *Tek-Cre* activity-mediated *Rosa* allele recombination was examined at E10.5. β-galactosidase activity was positive in all identifiable Pecam1^{+} endothelial cells, indicating complete recombination by E10.5. Sagittal sections containing pharyngeal arch and somite regions are shown. 3rd, 4th and 6th PAAs are numbered (III, IV and VI). DA, dorsal aorta; ISV, intersomitic vessel. Scale bars are 100μm.

Figure S5

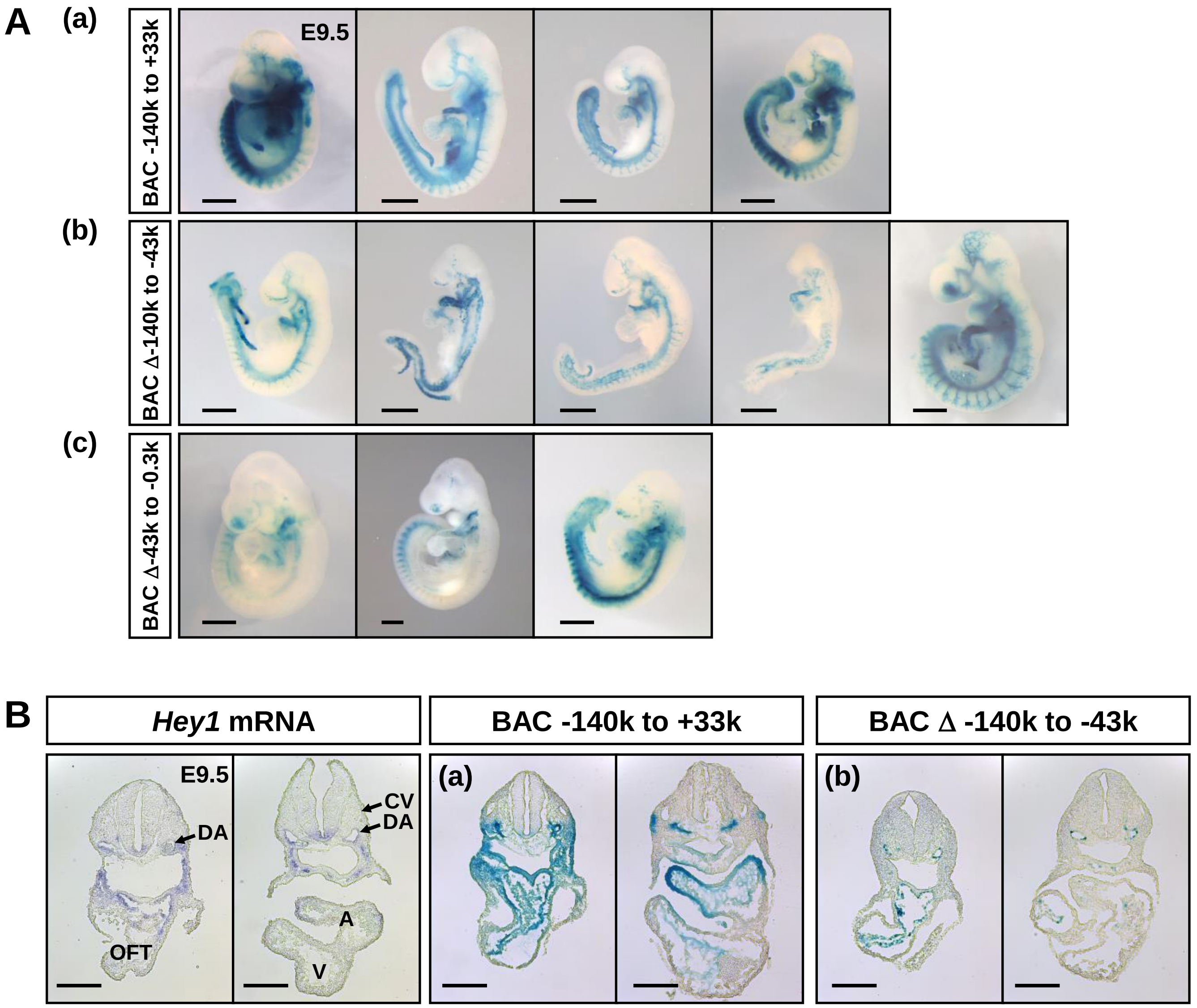

Fig. S5. (A) F0 transgenic mouse embryos in BAC-*LacZ* reporter analysis depicted in Fig. 3. β-galactosidase activity by the full-length BAC-*LacZ* reporter (a) reproduced endogenous *Hey1* mRNA expression in various embryonic tissues. Deletion of the -43k to -0.3k region diminished the activity in large caliber arteries (c), while that of the -140k to -43k region did not affect arterial reporter expression (b). Scale bars are 500μm. (B) Sections of the embryos from whole mount *in situ* hybridization and BAC-*LacZ* reporter analysis showed vascular endothelial expression of *Hey1* mRNA and *LacZ* reporters (a, b), respectively. A, atrium; CV, cardinal vein; DA, dorsal aorta; OFT, outflow tract; V, ventricle. Scale bars are 200μm.

Figure S6

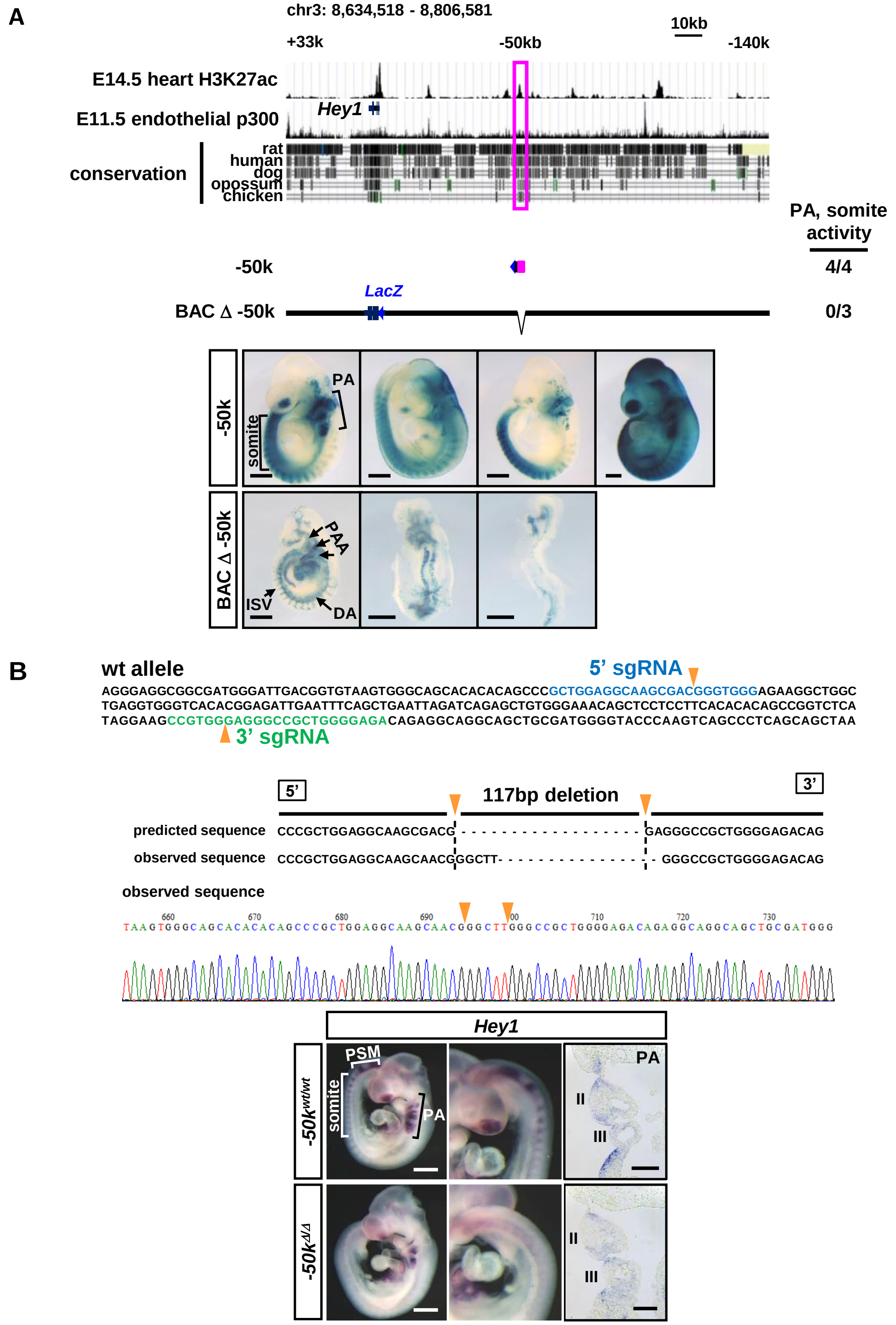

Fig. S6. (A) The H3K27ac ChIP-seq result using E14.5 heart and genomic conservation proposed mouse *Hey1* -50k region as an enhancer candidate. F0 transgenic mouse analyses revealed the 0.6k fragment in the -50k region as an enhancer for pharyngeal epithelium and somites. Scale bars are 500μm. (B) sgRNAs were designed at 5' and 3' regions of the -50k region. Orange triangles indicate Cas9 cleavage sites. Predicted and observed sequences by CRISPR/Cas9 genome editing are shown. *in situ* hybridization indicated that *Hey1* expression was decreased in the pharyngeal epithelium and somites of the -50k deletion homozygotes (*-50k*$^{\Delta/\Delta}$). DA, dorsal aorta; ISV, intersomitic vessel; PA, pharyngeal arch; PAA, pharyngeal arch artery; PSM, presomitic mesoderm. Scale bars in whole-mount views and sections are 500μm and 100μm, respectively.

Figure S7

A

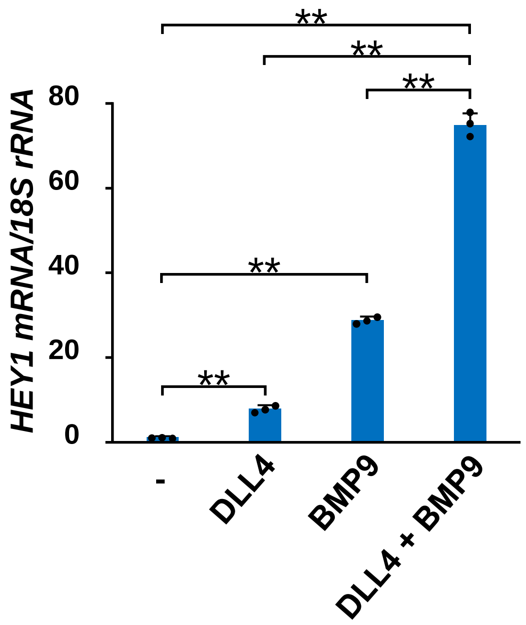

B

-532

ggctcccccgcctggctattcttctccattcatgcagccgcccagagcagccgcccagagcagccgccgccgccgccaccgt
cgccgccgccgtcgccgtccgtccggattggctggcggccggggcgccggcactttcccacggcccgcggcgcgtgcggctcac
cggcggcgctgcaggcgcggccctggcacacggccaggccaatccgggcgcgcggggtggggctgggcggggcggtggctat
cgccgggctggtgtcccgggccggccccccatttctgcccgccgccggcccaccgccgcccgcggcccccagccgaactcctgc
ggagccgacgcactgccgccgggcaggggcgggctccgggggcggggagcagccagcctcggcctccgctcgtcccgccccc
atgcggctgccaatctgcgcagcgaggcgccaattggccggggcacgcggttacccgggagccgtggccccgcccctgccccaa
cctctccgccttccccgccctcccccctatccATGGGAAGGGGCGCAGCGTGGGAAAGGGATGGTTGAGTTTT
AACCGGAGACTGAGCGTGAGTGGGATCAGTGTGCACGCACCTCCCGCAGCCGAGCGCTGAGCG
GCCACTGCAGTTAACTCCTCCTTGCCCGCCGCGCGACCCTCCTCGGAGCCCACGCTCCGCCACC

+168

red: essential Rbpj binding site **pink: putative Rbpj binding site**
green: GC-rich SMAD binding site

C

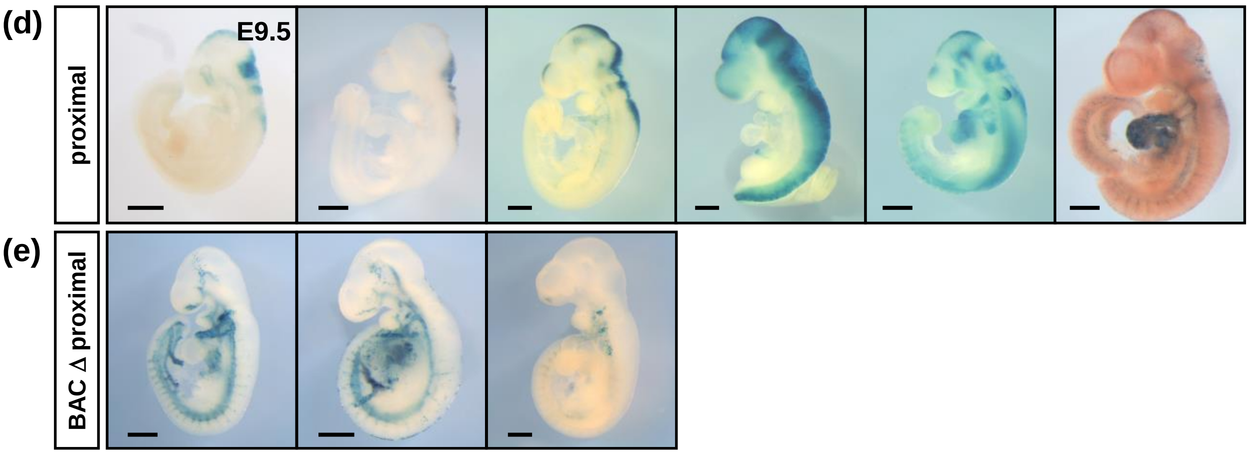

Fig. S7. (A) Realtime PCR analysis revealed synergistic induction of *HEY1* mRNA expression by Notch and ALK1 signaling. Human umbilical vein endothelial cells were cultured on DLL4-coated or non-coated dishes with or without the BMP9 treatment (1ng/ml) for 6 hr. **, P < 0.01. (B) Sequence of mouse *Hey1* proximal region used for luciferase and *LacZ* analyses. The arrow indicates a major transcriptional start site based on the information from the UC Santa Cruz genome browser. Consensus binding sites for Rbpj and SMAD are shown. A Rbpj binding site shown in red was mutated for luciferase and *LacZ* analyses. (C) F0 transgenic mouse embryos in *LacZ* reporter analysis depicted in Fig. 4B. The *Hey1* proximal region is insufficient to drive endothelial transcription and is dispensable for full-length BAC-*LacZ* activity in the vasculature. The first embryo for each reporter (d, e) is used in Fig. 4B. Scale bars are 500μm.

Figure S8

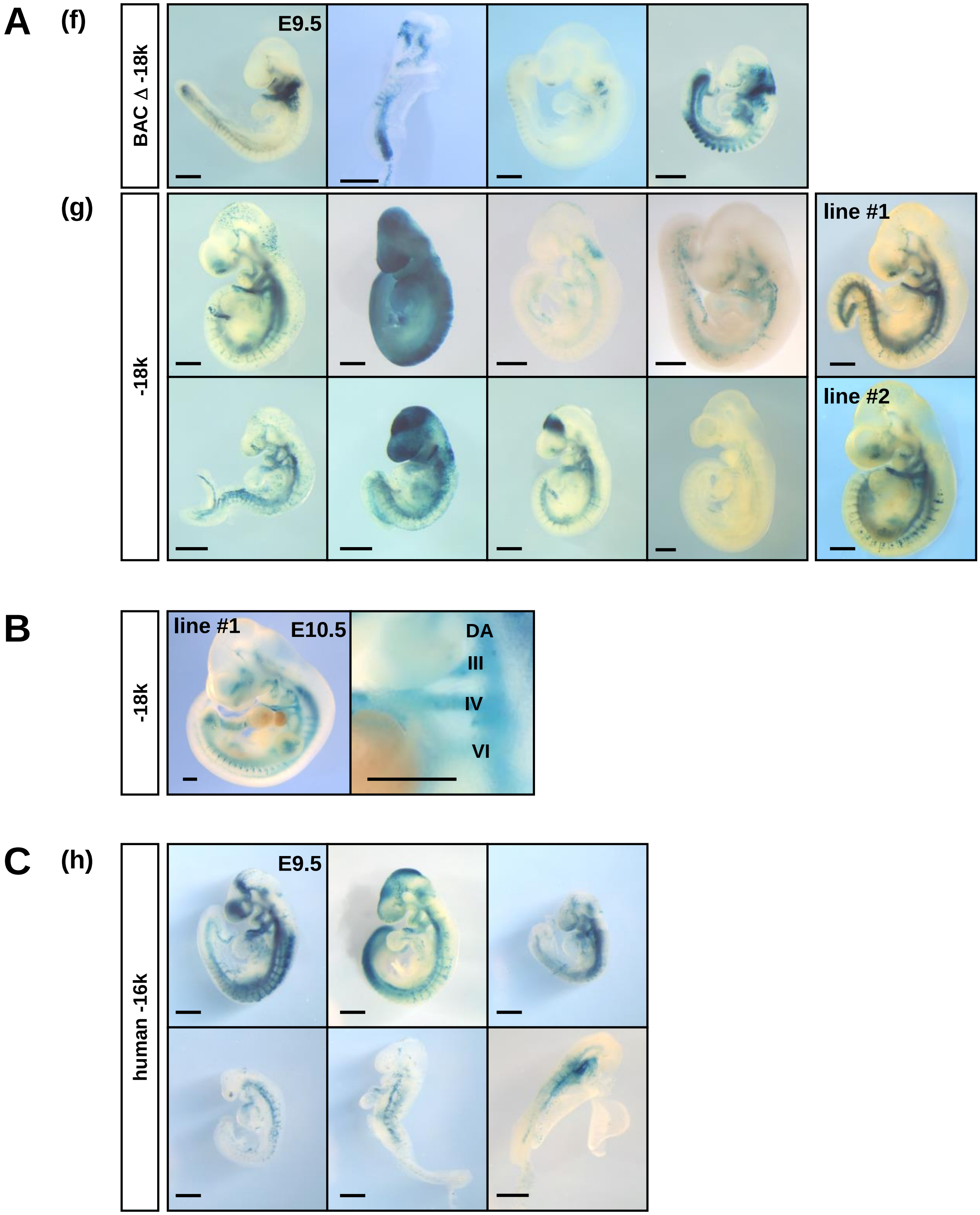

Fig. S8. (A) F0 transgenic mouse embryos in *LacZ* reporter analysis at E9.5 depicted in Fig. 5B. The BAC-*LacZ* reporter lacking the mouse -18k region (f) did not possess robust transcriptional activity in the vasculature, while the 18k region (g) drove endothelial reporter expression. The first embryo for each reporter is used in Fig. 5B. The results of the -18k-*LacZ* reporter lines (#1 and #2) are also shown. (B) Transcriptional activity of the -18k-*LacZ* reporter in 3rd, 4th and 6th PAAs (III, IV and VI) at E10.5 is shown using the line #1. (C) F0 *LacZ* reporter mouse embryos for the human -16k region depicted in Fig. 5B. The human -16k region (h) also induced endothelial reporter expression. The first embryo is used in Fig. 5B. DA, dorsal aorta. Scale bars are 500μm.

Figure S9

A **wt allele**

5’ sgRNA

CTGTGTAGCAAGGATGACCTTTGATTTATGATCCTCCTGCCTCCATTTCTCCAGTACCGACACTGCCAGCAGA
CAGCAAGTACCCCCATGCTGGGAATCCAACCCAGGGCTACACCTGCTAGGCAAGGACTCTACCAACTGAGCT
ACATTCCTGTCCCTGGAAGTCATTCTTGAGGGCTGGGGAACTTTCTCTTTTCTTAGATCTGTAAGCTATTTAGA
GCTAGGTTCTAGATGGTCTACCTTTGCACACCCAATTCAGTGCCGGGCTCACTCGGCA..........................TA
AAACCTCCAGCTTTATTGAGATACAATTCATATTCAATTCACTCACTTAAAATATGTGATTGGATCAATTTTAGT
GGTTTCCTACGTATGCACAACTACCTACTAGTGTTAGGTAATGGTAGAATATTTTCATTTTCTTTTCTTTTTTTTA
TTGAAACTTTTTTTAATTAGGTATTTTCTTCATTTACATTTCCAATGCTATCCCCCACTCCCCTACCCACCCACT
CCCACTTCTTGGCCCTGGTGTTCGCCTGTACTGAGGCATATAAAGTTTGCAAGACCAATGGGCCTCTCTTTCC
ACTGATGGCAAAATCTTGGTTGGTGGCATAGCTCAGTAACACAAGTGTGGGAGACCCTAGGTTCAATCTCCAA

3’ sgRNA

red: *mHey1* -18k region (1,590bp)

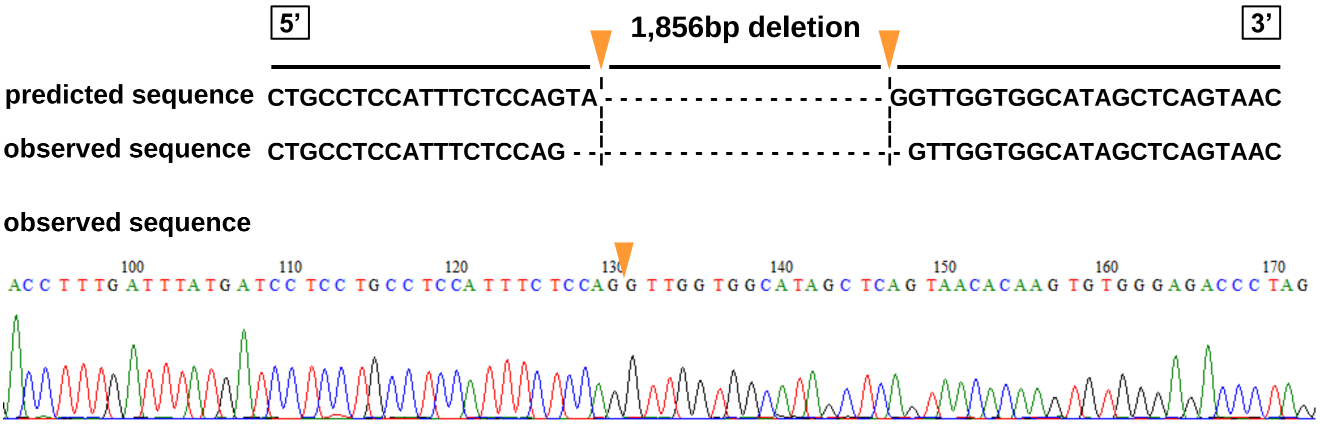

B

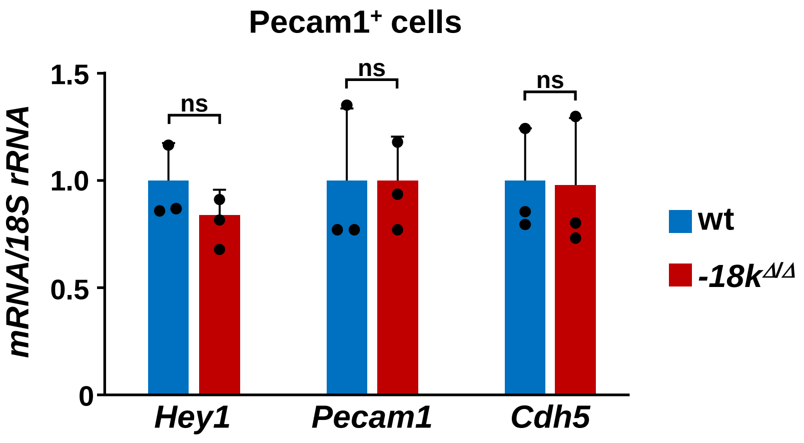

Fig. S9. (A) Two single guide RNAs (sgRNAs) were designed at 5’ and 3’ regions of the -18k region. Orange triangles indicate Cas9 cleavage sites. Predicted and observed sequences by CRISPR/Cas9 genome editing are shown. (B) *Hey1* mRNA expression in Pecam1^{+} endothelial cells was not significantly different between wild-type embryos and homozygotes for the -18k deletion (*-18k*$^{\Delta/\Delta}$). mRNA levels of *Pecam1* and *Cdh5* were analyzed as controls. ns, not significant.

Figure S10

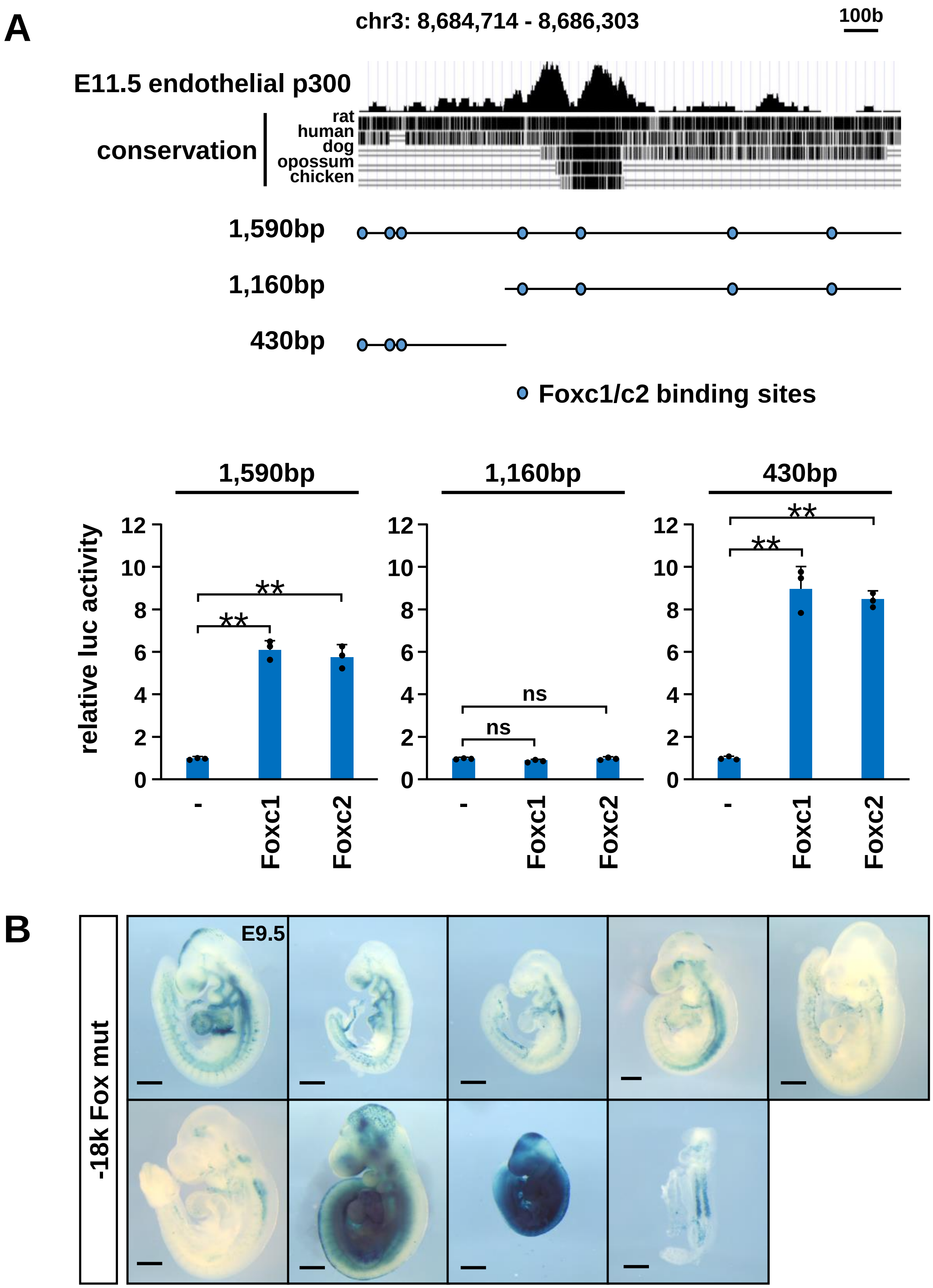

Fig. S10. (A) The p300 ChIP-seq result, genomic conservation, and Foxc1/c2 binding sites at the -18k region are shown. A 430bp fragment was necessary and sufficient for the transcriptional activation by Foxc1/c2. **, P < 0.01; ns, not significant. (B) F0 transgenic mouse embryos in *LacZ* reporter analysis depicted in Fig. 6D. The Foxc1/c2 binding sites are not essential for endothelial transcription. The first embryo is used in Fig. 6D. Scale bars are 500μm.

Figure S11

A

	Rbpj site-1		Rbpj site-2	
**mouse -18k**	**CGTGATGGGAA**	**TTGGAAACAAAGTCGCCAACAATAGGAGC**	**GTGGGAA**	**CCCC**
**human -16k**	**CGTGATGGGAA**	**TTGGAAACAAAGTCGCCAACAATAGGAGC**	**GTGGGAA**	**CCCG**
**chicken -5k**	**CGTGATGGGAA**	**TAGGAAACAAAGTCACAAACAATAGTAGC**	**GTGGGAA**	**CGCA**
**frog -8k**	**CGTGATGGGAA**	**TAGGAACCACAGCAGCAAACAATGGTAGC**	**GTGGGAA**	**CCCA**

B

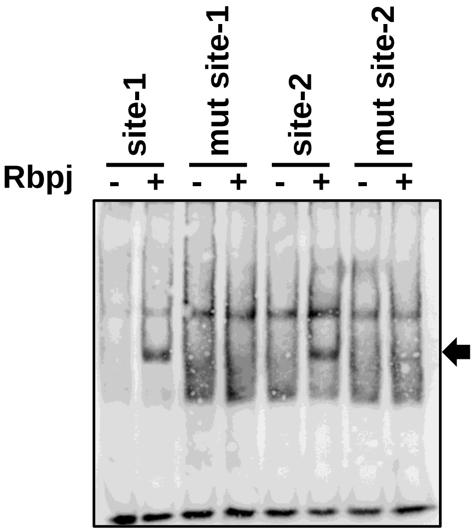

C

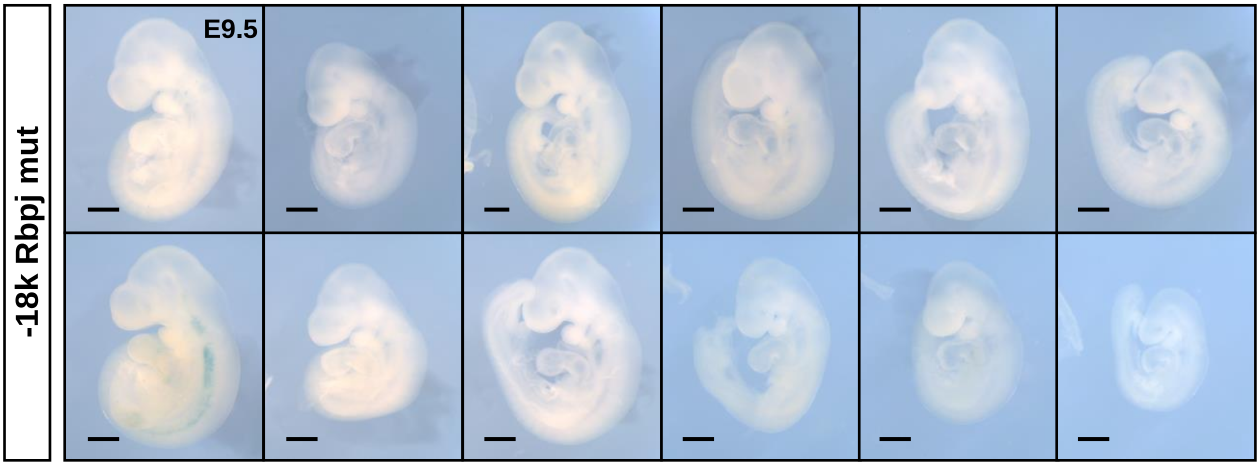

Fig. S11. (A) The mouse -18k region and its comparable genomic regions of the human, chicken and frog contain two conserved Rbpj binding sites. (B) Electromobility shift assay verified the binding of Rbpj to these sites (arrow), which was lost by the mutations used for luciferase and *LacZ* analyses. (C) F0 transgenic mouse embryos in *LacZ* reporter analysis depicted in Fig. 6D. The -18k region with mutated Rbpj binding sites did not induce *LacZ* reporter expression in transgenic embryos. Results of β-galactosidase reaction of all *LacZ* genotyping positive embryos are shown. The first embryo is used in Fig. 6D. Scale bars are 500μm.

Table S1

Single guide RNA target sites of the *Hey1* locus

target	site-1 (5' to 3')	site-2 (5' to 3')
exon 2, 3 and 4	TTGGCGGCGTCCGACCGATCCGG	GGCTTTGCGTGCTCGGCGTGGGG
-50k region	GCTGGAGGCAAGCGACGGGTGGG	TCTCCCCAGCGGCCCTCCCACGG
-18k region	TCTGCTGGCAGTGTCGGTACTGG	ACTGATGGCAAAATCTTGGTTGG

Genotyping PCR primers for *Hey1* wild type, floxed and deleted alleles

name	5' to 3'
F1	CGAGAATGGGTGAGTTGG
R1	GGGGACATGGAACACAGC
R2	GACACGGGCTTCAGTCTCTC

Primers for realtime PCR analysis

gene	forward (5' to 3')	reverse (5' to 3')
Hey1	CTGAGCTGAGAAGGCTGGTACC	ACCCCAAACTCCGATAGTCC
EGFP	GGACGACGGCAACTACAAGA	CTCGATGTTGTGGCGGATCT
Cdh5	TCAACGCATCTGTGCCAGAGAT	CACGATTTGGTACAAGACAGTG
Pecam1	GGTGCATGGCGTATCCAAG	TGGAGGTCTTATCTATCCTTCGC
Cxcr4	AAACCTCTGAGGCGTTTGGT	GCCGACTATGCCAGTCAAGA
Efnb2	AGGAATCACGGTCCAACAAG	GTCTCCTGCGGTACTTGAGC
Gja4	AGTGCCTCAGACCCTTACC	GAGTGACATTAGCCCCAGAT
Gja5	AGAGCCTGAAGAAGCCAACT	GGCGTGGACACAAAGATGA
Dll4	CCCTCTGCAGTTGCCCTTCA	GCTCGTCTGTTCGCCAAATC
Jag1	TCGCATCGTACTGCCTTTCA	TACTCGAAGTGGGCAATCCC
Hey2	GAGAAGACTAGTGCCAACAGC	GCATGGGCATCAAAGTAGCCT
Ephb4	GGTCAGCGCTCTGGACAAGATG	AGCCGAATCCAGCCGCTGCAA
18S rRNA	GTCTGTGATGCCCTTAGATG	AGCTTATGACCCGCACTTAC

Movies S1 and S2

Movie S1. MicroCT analysis of the control (*Hey1*$^{wt/fl}$;*Tek-Cre*$^{+}$) embryo at E18.5 showed normal thoracic great vessel structure.

Movie S2. MicroCT analysis of the endothelial cKO (*Hey1*$^{fl/\Delta}$;*Tek-Cre*$^{+}$) embryo at E18.5 showed the right-sided aortic arch.
